# Immunometabolism of Liver Xenotransplantation and Prospective Solutions

**DOI:** 10.1002/advs.202407610

**Published:** 2025-02-06

**Authors:** Shoulong Deng, Yi Zhang, Shasha Shen, Chongyang Li, Chuan Qin

**Affiliations:** ^1^ National Center of Technology Innovation for Animal Model, National Human Diseases Animal Model Resource Center, National Health Commission of China (NHC) Key Laboratory of Comparative Medicine, Institute of Laboratory Animal Sciences Chinese Academy of Medical Sciences and Comparative Medicine Center Peking Union Medical College Beijing 100021 China; ^2^ Department of Medicine Panzhihua University Sichuan 61700 China; ^3^ Institute of Animal Sciences Chinese Academy of Agricultural Sciences Beijing 100193 China

**Keywords:** antigens, immunometabolism, liver, Treg, xenograft rejection

## Abstract

End‐stage liver diseases, such as hepatocellular carcinoma or acute liver failure, critically necessitate liver transplantation. However, the shortage of available organ donors fails to meet the rapidly growing transplantation demand. Due to the high similarity of liver tissue structure and metabolism between miniature pigs and humans, xenotransplantation of pig livers is considered as a potentially viable solution to organ scarcity. In the 2024, teams from China first time have successfully transplanted a genetically modified Bama miniature pig liver into a clinically brain‐dead man lasting for 10 days. This milestone in human xenotransplantation research not only confirms the feasibility of clinical application of xenotransplantation, but also underscores the daunting and protracted nature of this pathway. Despite advanced gene‐editing technologies theoretically circumventing the occurrence of most transplant rejection reactions, patients still face challenges such as chronic immune rejection, coagulation disorders, and thrombotic microangiopathy after receiving xenografts. Moreover, prolonged use of immunosuppressive drugs may induce irreversible immune dysfunction, leading to opportunistic infections and metabolic disorders. This article compares the similarities and differences in livers between humans and pigs, summarizes the immunometabolism of xenotransplantation based on current findings, and provides research perspectives on pre‐transplantation and post‐transplantation strategies for prolonging the survival time of xenografts.

## Graphical abstract

After xenotransplantation, pig livers will replace those of human patients to maintain the stability of metabolism and immune system. The incompatibility of immunometabolism between humans and pigs induces rejection, inflammation, coagulation dysfunction, and liver failure. The profile of immunometabolism pre‐transplantation and post‐transplantation is meaningful for the establishment of donor pigs and the achievement of immune tolerance. Adapted with permission from BioRender. Kelai, W. (2024).

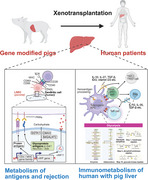

## 1 Introduction

The liver is an organ with complex structures and functions. The pig's liver shares high structural, cellular compatibility, and physiological metabolic similarities with the human one,^[^ ^1^, ^2^ ^]^ making it a suitable model for xenotransplantation into humans. After pig liver xenotransplantation, there are numerous immunological barriers, with graft failure primarily attributed to interspecies incompatibility of functional glycoproteins or proteins, immune rejection responses, and coagulation dysfunction.^[^ ^3^ ^]^ Proteins and enzymes derived from pigs are incompatible with the human system, with over 100 proteins produced by the pig liver potentially not functioning effectively in humans. Wild‐type pig liver transplants typically undergo early antibody‐ and complement‐mediated rejection (intravascular thrombosis, hemorrhagic necrosis, endothelial cell damage, IgM, IgG, C3 complement deposition), acute cellular rejection, and chronic rejection.^[^ ^4^ ^]^ Meanwhile, the differences in the expression and functional regulation of coagulation proteins between human and porcine hepatocytes, the inactivation of pig platelet regulators, and the hyperfunction of von Willebrand factors (vWFs), lead to platelet activation, aggregation, and/or phagocytosis, causing severe thrombocytopenia, bleeding, and thrombosis.^[^ ^5^ ^]^ Therefore, deciphering the differences in the immunometabolism between human and pig livers, and establishing xenotransplant‐specific immune tolerance, is crucial for the success of liver xenotransplantation.

Unlike the conventional definition of “immunometabolism,” the immunometabolism of xenotransplantation places more emphasis on the following two points: 1) the metabolic process of xenoantigens production in donor pigs and related immune rejections after transplantation (immunometabolism of antigens); 2) the regulatory role of human metabolism on the strength of immune rejection induced by xenoantigens. Both of them are important for the success of xenotransplantation. Exploration of the metabolic process in donor pigs is helpful in finding gene editing targets to produce low or nonimmunogenic xenotransplantation organs. At the same time, understanding the metabolic changes in human patients after xenotransplantation can help to develop novel strategies with low side effects to induce immune tolerance and control immune responses. This paper will comprehensively illustrate the findings related to these two aspects and introduce potential solutions for further studies.

## 2 Feasibility of Pig‐to‐Human Liver Xenotransplantation

There is a huge gap between the number of patients waiting for a liver transplant and the number of available livers for transplantation. According to the Global Observatory for Donation and Transplantation, the current supply could only meet ≈10% of the demand for liver transplantation.^[^ ^6^ ^]^ Many patients die every day because of failure to obtain a suitable organ. To solve the shortage of organs, several approaches have been proposed, like 1) increasing donor donation, 2) “bioengineering” of transplantable organs (tissue engineering), and 3) the use of organs from animals, known as xenotransplantation. However, the potential success of increasing donor donation is limited, because deaths resulting in transplantable organs (brain death and cardiac death) only account for a tiny fraction of mortalities (less than 1%).^[^ ^7^ ^]^ Whereas, tissue engineering, which applies stem or progenitor cells to populate 3D‐printed scaffolds to construct human organs, has not yet been successful in large animal models and is hampered by many hurdles, including technical difficulties, high costs, safety concerns, and ethical issues.^[^ ^7^ ^]^

At present, transplantation of animal organs to humans seems to be the most applicable clinical option. As early as the 1960s, Tom Starzl and colleagues have conducted a series of experiments transplanting chimpanzee livers into humans.^[^ ^8^ ^]^ Subsequently, researchers continued to explore the use of more readily available baboons as liver donors. In the 1990s, they successfully transplanted livers from baboons to humans and extended one patient's survival to 70 days.^[^ ^8^ ^]^ However, most primates are endangered species, making their availability low.^[^ ^7^ ^]^ In addition, there are ethical concerns in using primate organs, and safety problems that recipients may be infected with zoonosis from primate donors.^[^ ^7^ ^]^ As a result, using primates for xenotransplantation has been progressively prohibited. These issues have led the field of transplantation to seek another, less problematic source animal for xenotransplantation.

**Figure 1:**
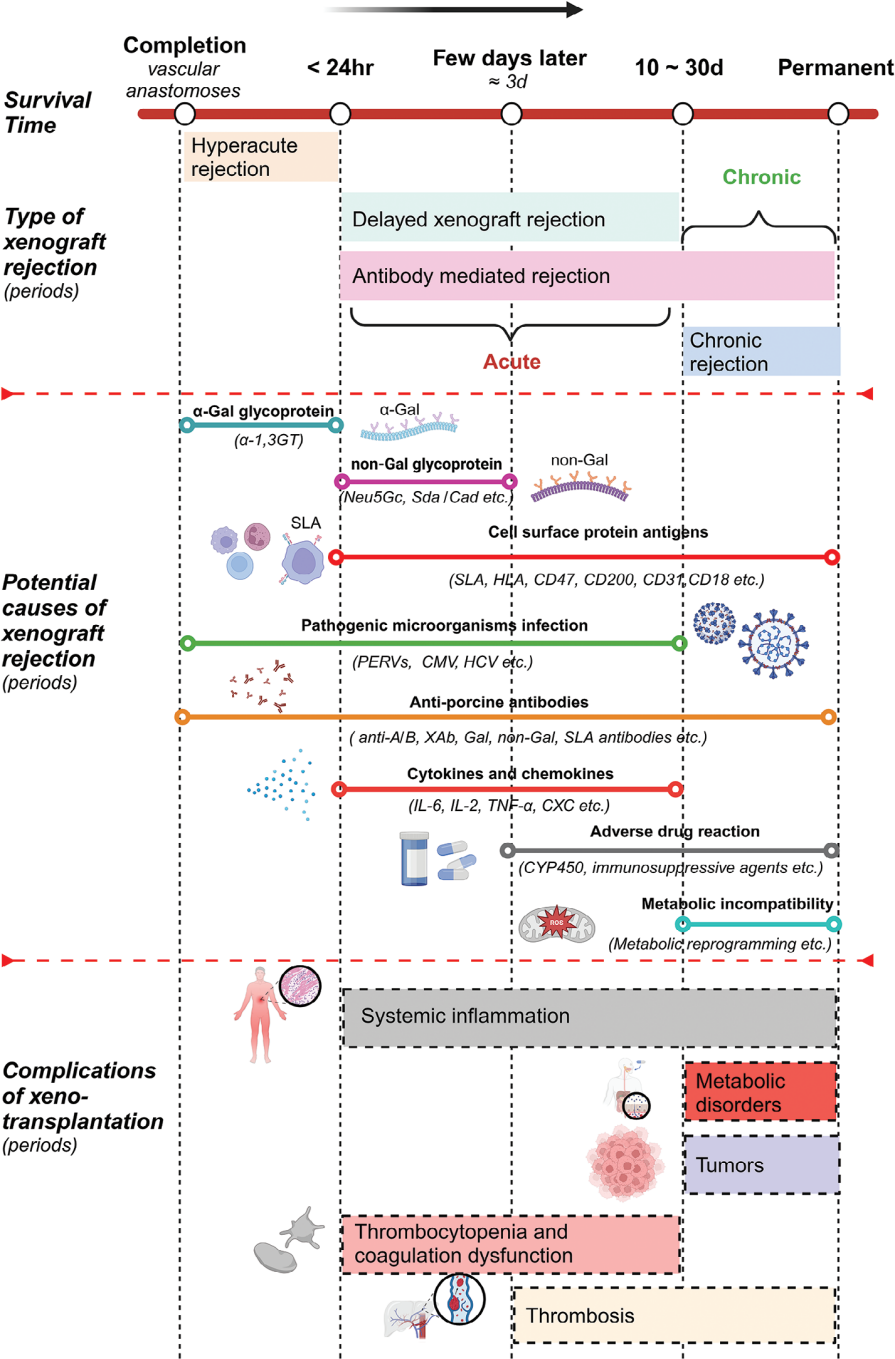
Schematic timeline showing the phases of immune rejection of liver xenotransplantation from pig to primates. Pig‐to‐primate liver transplantation is subject to vigorous immunologic rejection involving both innate and adaptive immune responses. The high levels of natural antibodies (NAbs) to Gal in human serum, which responsible for hyperacute rejection (HAR) within hours after transplant, are a major early obstacle to xenotransplantation. Following HAR, NAbs against other pig specificities, such as non‐Gal and SLAs antigens, mediate a more delayed type of vascular xenograft rejection, termed “delayed xenograft rejection (DXR)” or “acute humoral xenograft rejection (AHXR),” which occurs over days to weeks, rather than minutes to hours, as is the case with HAR. In addition, antibody‐ and complement‐independent cellular xenograft rejection (CXR) also appears in the period of DXR. CXR is mediated by cells involved in the innate and/or the adaptive immune system, such as monocytes, macrophages, natural killer (NK) cells, neutrophils, dendritic cells (DCs), T cells, and B cells. The cytokines or chemokines released by these cells are related with the cytotoxicity, inflammation, oxidative injury and apoptosis in xenografts. Endothelium injury from AHXR and CXR, exposes tissue factor (TF) and collagen, which results in the development of thrombotic microangiopathy in the graft. Severe complications, such as metabolic disorders or tumors, will arise if the xenograft survives long‐term. It is related to the low‐amplitude immune responses, termed “chronic rejection,” attributed to biological incompatibility between pigs and NHPs/humans due to the specificity of liver metabolism and synthesis. Abbreviations: PERVs, porcine endogenous retroviruses; CMV, cytomegalovirus; HCV, hepatitis C virus. Adapted with permission from BioRender. Kelai, W. (2024).

Most researchers agree that pigs are the most appropriate source animals for xenotransplantation due to the similarities in size, availability, reproductive and physiological characteristics.^[^ ^7^ ^]^ By knocking out the growth hormone receptor (GHR) gene in pigs, the growth rate of pig organs intended for xenotransplantation could be controlled.^[^ ^9^ ^]^ The liver of GHR‐KO “miniature pigs” exhibits a size and anatomical structure nearly identical to that of the human liver.^[^ ^9^, ^10^, ^11^ ^]^ The liver serves as the primary site for the breakdown and synthesis of organic compounds, including carbohydrates, proteins, and lipids, playing an essential role in maintaining and regulating metabolic balance within the body. Most plasma proteins associated with immune rejection and coagulation functions, such as albumin, globulins, coagulation factors, and transport proteins, are synthesized in the liver.^[^ ^10^ ^]^ Animal models of miniature pigs developed through gene editing technology have been widely employed in researching human liver metabolic diseases to explore the mechanisms underlying the occurrence and development of liver metabolic diseases. The liver of miniature pigs used for organ transplantation shows no significant differences in total carbohydrates, total proteins, and total lipids compared to the human liver.^[^ ^9^, ^10^, ^12^, ^13^, ^14^, ^15^, ^16^, ^17^, ^18^ ^]^ Direct transplantation of wild‐type pig livers could induce acute or hyperacute immune rejection (**Figure** **1**).^[^ ^19^ ^]^ Donor pig livers constructed by knocking out genes of xenoantigens (α‐1,3‐galactosyltransferase (GGTA1) CMP‐Neu5Ac hydroxylase (CMAH)β‐1,4‐N‐Acetyl‐galactosaminyltransferase 2 (B4GaINT2), etc.) or introducing human genes coding cell surface receptors (hCD46, hCD47, hCD55, etc.), could effectively alleviate or reduce the intensity of the immune response (**Table** **1**).^[^ ^7^, ^19^ ^]^ This was followed by encouraging results in living patients, including the first pig‐to‐human liver transplantation with more than 10 days survival in 2024 (Figure 1).^[^ ^20^ ^]^ If successful to reveal the mechanism of immunometabolism of rejection, therefore, then xenotransplantation could provide a near‐term solution for the human organ shortage.

**Table 1:** Survival time of gene editing xenogeneic liver.

Year	Genetic modifications	Recipient	Recipient number	Type	Immunosuppression	Cause of death	Survival time	Refs.
2024	GTKO/CMAH‐KO/B4GALNT2‐KO/hCD46/hTM	human	1	Heterotopic	ATG + CVF + TAC + aCD40mb + aCD20mb + MPS	Unknown	10 d	[20]
2020	PERV‐KO/3‐KO/9‐TG	Rhesus monkey	1	Heterotopic	ATG + CVF + TAC + aCD40mb + aCD20mb + MPS	Patchy necrosis/interstitial hemorrhage/thrombotic microangiopathy/inflammatory damage	26 d	[19, 243]
2017	GTKO/hCD47	Tibetan macaques	6	Heterotopic	ATG + CVF + FK + MMF + Medrol	Pulmonary hemorrhage/edema/infection/renal failure/cytokines elevation bring out liver xenogfart damage	Max.(14 d)	[19, 244]
2017	GTKO	Baboon	4	Orthotopic	ATG + CVF + Tac + Cs + aCD40mAb	Portal vein thrombosis/minimal inflammation/thrombotic microangiopathy	Max. (29 d)	[19, 245]
2016	GTKO	Baboon	1	Orthotopic	ATG + CVF + Belatacept + Tac + Cs	Focal hepatic necrosis/mild congestion	25 d	[19, 246]
2016	GTKO	Baboon	6	Orthotopic	ATG + CVF + Tac + Cs	Thrombotic microangiopathy/inflammation	Max. (7 d)	[19, 247]
2015	GTKO	Tibetan macaques	3	Heterotopic	ATG + CVF + Tac + MMF + anti‐CD154 mAb	Pulmonary edema/infection	Max. (14 d)	[19, 248]
2014	GTKO	Baboon	3	Heterotopic	ATG + Cs + Tac + CVF	Thrombotic microangiopathy/sepsis	Max. (15 d)	[19, 249]
2012	GTKO	Baboon	3	Orthotopic	ATG + CVF + anti‐CD154mAb + AZA + LoCD2b + Tac + Cs	Uncontrolled bleeding/enterococcal infection/no rejection	6, 8, 9 d	[19, 250]
2010	GTKO/hCD46	Baboon	10	Orthotopic	ATG + Tac + MMF + Cs	Size‐mismatch in donor liver and recipient abdomen/profound thrombocytopenia liver toxic (clodronate)	Max. (7 d)	[19, 251]
2005	hCD55/hCD59/HT	Baboon	9	Orthotopic	CyP + Dacluzimab + Cs + Rituximab + CsA + MMF	Thrombosis/IVC/ARF/APF/no HAR (hCD55/hCD59/HT) multiorgan failure/DIC	Max. (16 h)	[19, 252]
2000	hCD55	Baboon	5	Orthotopic	CyA + CyP + MPS + splenectomy	No deposits of C3 or C5b‐9/no HAR (hCD55) endothelial deposits of IgG, IgM, C3, C4	Max. (8 d)	[19, 253]

CyP: cyclophosphamide; CyA: cyclosporine A; HT: H‐transferase; DIC: disseminated intravascular coagulation; IVC: inferior vena cava; ARF: acute renal failure; APF: acute pulmonary failure; MMF: mycophenolate mofetil; Cs: corticosteroids; CsA: cyclosporine; LoCd2b: rat antiprimate CD2 IgG2b; MPS: methylprednisolone; Tac: tacrolimus. PERV: porcine endogenous retrovirus; hTM: human thrombomodulin; HT: human α‐1,2‐fucosyltransferase; GTKO/CMAH‐KO/B4GALNT2‐KO/PERV‐KO: knockout porcine genes of GGTA1, CMAH, B4GALNT2 and PERV; hCD46/hTM/hCD47/hCD55/hCD59/HT: knockin human genes of CD46, TM, CD47, CD55, CD59 and HT; 3‐KO/9‐TG: knockout 3 porcine genes and knockin 9 human genes.

## 3 Pathology of Liver Xenograft Rejection

**Figure 2:**
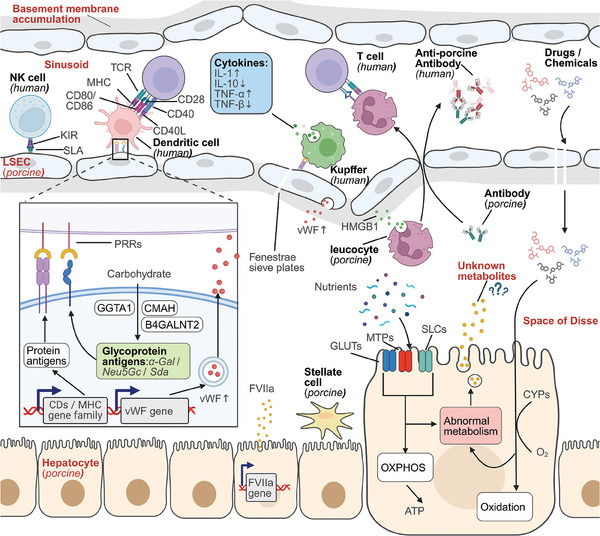
Immunometabolism of cells in xenogeneic liver. Acute and hyperacute immune rejection in liver xenotransplantation is primarily triggered by protein or glycoprotein xenoantigens present on the surface of liver sinusoidal endothelial cells (LSECs), hepatocytes, and donor‐derived leukocytes. These cells are extensively distributed in the hepatic sinusoids and space of Disse (lymphatic spaces). They generate rejection‐related cell surface antigens through the expression of porcine‐specific genes such as GGTA1, CMAH, and B4GALNT2. In the preparation stage before transplantation, washing or cleaning the donor liver could remove most of porcine leukocytes, but LSECs and hepatocytes could not be eliminated. Following vascular anastomoses, recipient‐derived immune cells flow into the hepatic vasculature and ultimately reach the hepatic sinusoids, where they recognize LSECs, residual leukocytes, or porcine antibodies, initiating xenoantigen presentation and expression of proinflammatory cytokines. Damaged LSECs expose tissue factor (TF), which binds with activated factor VII (FVIIa) and initiates thrombin generation. Accompanied by that, porcine von Willebrand factor (vWF) recognizes human platelet glycoprotein 1b (GP1b) and activates the aggregation of platelets, ultimately leading to coagulation dysfunction in the recipient. Continuous damage to hepatic endothelial cells by the recipient's immune system may result in sinusoidal obstruction syndrome (SOS), hepatic vein or artery thrombosis. In addition, there is no clinical report to prove that medications or nutrients intake by humans are fully metabolized by the xenogeneic liver. The unknown metabolites produced by porcine liver potentially result in metabolic syndromes or graft failure. Abbreviations: HMGB1, high mobility group protein B1; TCR, T cell receptor; KIR, killer cell immunoglobulin‐like receptor; PRRs, pathogen recognition receptors; GLUTs, glucose transporters; MTPs, microsomal triglyceride transporters; SLCs, solute carrier transporters. Adapted with permission from BioRender. Kelai, W. (2024).

Although the metabolic patterns of pig and human livers are highly similar, direct xenotransplantation of pig‐to‐human liver organs remains challenging.^[^ ^19^ ^]^ Hyperacute immune rejection (HAR) in liver xenotransplantation usually occurs within 24 h after vascular anastomoses. Delayed xenograft rejection (DXR) and chronic rejection follow HAR and cause graft destruction that lasts for a few weeks to months (Figure 1). The main obstacle lies in the species‐specific carbohydrates, glycoproteins, or proteins produced by pig liver.^[^ ^19^ ^]^ These antigens are widely present on the surface of liver sinusoidal endothelial cells (LSECs), hepatocytes, and donor‐derived leukocytes in the hepatic sinusoids and space of Disse (lymphatic spaces) (**Figure** **2**). Based on structure characteristics, xenogeneic antigens could be classified as glycoprotein antigens (Gal antigens and non‐Gal antigens), and protein antigens (SLA, GABARAPL1, HMGB1, etc.). In the pig liver, cells highly express porcine‐specific genes such as GGTA1, CMAH, and B4GALNT2, and allow them to act as crucial metabolic enzymes to maintain the required immunometabolism, nutrients, and medications metabolism. However, the metabolites synthesized by porcine‐specific enzymes could not be utilized by humans or other primates. When they are present on the cell surface, the immune system of the transplantation recipient will recognize them as “foreign substances.” In the preparation stage before transplantation, washing or cleaning the donor's liver could remove most of the porcine‐specific metabolites and leukocytes, but LSECs and hepatocytes could not be eliminated.^[^ ^21^ ^]^ Following vascular anastomoses, recipient‐derived immune cells flow into the hepatic vasculature and ultimately reach the hepatic sinusoids, where they recognize LSECs, residual leukocytes, or porcine antibodies, initiating xenoantigen presentation and expression of proinflammatory cytokines (Figure 2). Damaged LSECs expose tissue factor (TF), which binds with activated factor VII (FVIIa) and initiates thrombin generation. Accompanied with that, porcine vWF recognizes human platelet lycoprotein 1b (GP1b) and activates the aggregation of platelets, ultimately leading to coagulation dysfunction in the recipient (Figure 2). Continuous damage to hepatic endothelial cells by the recipient's immune system may result in sinusoidal obstruction syndrome, hepatic vein or artery thrombosis. In addition, there is no clinical report to prove that the xenogeneic liver fully metabolizes medications or nutrients intake by humans. The unknown metabolites produced by porcine liver potentially result in metabolic syndromes or graft failure. To better explore the methods of overcoming immunological destruction caused by xenoantigens, it is necessary to clarify the metabolism processes they are involved in.

## 4 Immunometabolism of Potential Liver Xenotransplantation Antigens

### 4.1 Gal Carbohydrate Antigens

α‐1,3‐Galactose (Gal) plays a pivotal role in the HAR reactions of xenotransplants. Gal epitopes are extensively distributed in various porcine tissues, including endothelial cells, proximal tubular epithelial cells, epithelial cells covering the bronchioles of pulmonary alveoli, hepatocytes, bile duct epithelial cells, pancreatic ducts, fetal islets (absent in adult islets), and the deep layers of the skin (**Table** **2**). It is estimated that porcine endothelial cells (SECs) express ≈10^6^ Gal epitopes per cell.^[^ ^14^ ^]^ The synthesis of Gal antigen epitopes relies on α‐1,3‐galactosyltransferase (α‐1,3GT). α‐1,3‐Galactosyltransferase catalyzes the transfer of α‐galactose from UDP‐galactose to *N*‐acetyllactosamine, generating α‐1,3‐galactose.^[^ ^14^ ^]^ During the evolution of higher animals (humans, primates, and Old World monkeys), the gene encoding α‐1,3‐galactosyltransferase, known as GGTA1, underwent mutations and lost its activity.^[^ ^19^, ^22^ ^]^ It could only encode a truncated, nonenzymatic form of protein resembling α‐1,3‐galactosyltransferase but lacking the C‐terminal catalytic domain.^[^ ^14^ ^]^ This mutation renders higher animals incapable of producing α‐1,3‐galactose, forming a unique rejection response system against foreign microbes and antigens (Figure 2).

**Table 2:** Summary of antigens inducing immune rejections after xenotransplantation.

Porcine antigens	Type	Major located cells	Distribution characteristics	Crucial genes	Rejection types	Immunometabolism after transplantation	Potential complications	Refs.
α‐1,3‐Galactose	Carbohydrates (glycoproteins/glycolipids)	Endothelial cells/PBMCs/RBCs	78% of endothelial cells contain	GGTA1	HAR	Bind with high levels of NAbs to Gal in human serum	Liver failure/death	[23]
Neu5Gc	Carbohydrates (glycans/ glycoproteins/ glycolipids)	Endothelial cells/PBMCs/RBCs	12% in liver, 40% in spleen, 25% in lung	CMAH	DXR/AHXR/CXR	Bind with Nabs to Neu5Gc in human serum; induce the enrichment of effector T cells	Focal ischemia and diffuse intravascular coagulation	[254]
Sda/Cad	Carbohydrates (glycans/glycoproteins)	RBCs/PBMCs/vascular endothelial cells	Expressed in almost all erythrocytes	B4GALNT2	DXR/AHXR	Bind with NAbs to Sda in human serum	Hemolytic reaction/hemorrhage/thrombotic microangiopathy	[28]
SLA I	Proteins (swine leukocyte antigens)	All nucleated cells	Highest allele frequency in population: SLA‐3*0502 42.35%	SLA I	DXR/CXR	Present peptides to CD8+ cytotoxic T cells and interact with NK cells; activation of B and T cell proliferation	Inflammation/tissue damage/immune cell infiltration	[60, 255]
SLA II	Proteins (swine leukocyte antigens)	Antigen‐presenting cells (dendritic cells, macrophages, b cells)	Highest allele frequency in population: SLA‐DQB1*0801 58.72%	SLA II	DXR/CXR	Present exogenous peptides to CD4+ helper T cells; activation of B and T cell proliferation	Cytokines elevation/inflammation/cell death in grafts/immune cell infiltration	[60, 255]
CD46	Proteins (cluster of differentiation antigens)	Circulating cells (PBMCs/RBCs)	Fivefold more in peripheral blood than in liver	pCD46	HAR/DXR	Regulate T cell‐induced inflammatory reaction; link innate and adaptive immune responses	Thrombocytopenia/blood coagulation dysfunction/inflammation	[19, 256]
CD47	Proteins (cluster of differentiation antigens)	Macrophages/Kupffer cells	Abundantly expressed on monocytes/macrophages	pCD47	DXR/CR	Involve in Kupffer cell mediated phagocytic dysregulation	Thrombocytopenia/bleeding/ischemia	[19, 25]
CD11b/CD18	Proteins (cluster of differentiation antigens)	Macrophages/Kupffer cells	Abundantly expressed on monocytes/macrophages	pCD11b/pCD18	DXR/CR	Regulate platelet binding and phagocytosis by Kupffer cells	Diffuse intravascular coagulation/thrombocytopenia	[19, 25]
vWF	Proteins (coagulation‐related antigens)	Endothelial cells	Two‐ to fivefold elevation during the liver endothelial injury	pvWF	DXR/CR	Bind to glycoprotein 1b (GP1b) to activate platelets, which then bind to fibrinogen, leading to platelet aggregation and adherence to the endothelium	Thrombogenesis/coagulation dysfunction/systemic inflammation	[19, 243]
GABARAPL1	Proteins (autophagy‐related proteins)	vascular endothelial cells/antigen‐presenting cells (dendritic cells, macrophages, b cells)	47% of endothelial cells contain	pGABARAPL1	DXR/CXR	Regulating the transcription of SLA and non‐Gal antigens through diverse pathways	Focal hepatic necrosis/mild congestion	[76]
COX2	Proteins (prostaglandin G/H synthase family)	Endothelial cells	20% of endothelial cells contain	pCOX2	DXR/CXR	Dysregulation of COX2 affects accumulation of PG, homeostasis, inflammation, and cancer	Inflammation/tumor/tissue damage/thrombogenesis	[81]
HMGB1	Proteins (high mobility group box protein family)	Endothelial cells/monocytes/macrophages/dendritic cells	Moderate expression is present in all organs (except lymphoid tissue).	pHMGB1	DXR/CXR	Bind to TLRs on macrophages; activate NF‐κB and IRF pathways; induce the production of proinflammatory cytokines	Early liver damage/inflammation/cellular damage	[97]
PERVs	RNA (infection‐related)	All nucleated cells	Infect certain human cells	PERVs	HAR/DXR	Induce lymphoma, leukemia, and immunodeficiency in their infected hosts	Tumor/liver failure/death	[257]

PBMCs: peripheral blood mononuclear cells; RBCs: red blood cells; pCD46: porcine CD46 coding gene; pCD47: porcine CD47 coding gene; pCD11b/pCD18: porcine CD11b/CD18 coding gene; pvWF: porcine vWF coding gene; pGABARAPL1: porcine GABARAPL1 coding gene; pCOX2: porcine COX2 coding gene; pHMGB1: porcine HMGB1 coding gene; HAR: hyperacute rejection; DXR: delayed xenograft rejection; AHXR: acute humoral xenograft rejection; CXR: cellular xenograft rejection; CR: chronic rejection; Nabs: natural antibodies; PG: prostaglandins; IRF: interferon regulatory factor.

**Figure 3:**
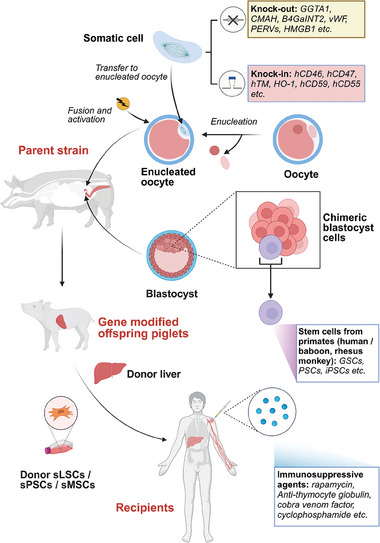
Current techniques of genetic modification and embryo engineering have been made in pigs to facilitate pig‐to‐human liver transplantation. Two strategies are currently being developed as a means for xenotransplantation. 1) Using gene editing and nuclear transfer techniques to eliminate xenoantigens such as GGTA1, CMAH, B4GALNT2, and HMGB1, or to introduce humanized protein genes related to immunosuppression and coagulation functions, such as hCD46, hCD47, hTM, and HO‐1. Donor pig livers produced using gene editing techniques have already been successfully used in human liver transplants. However, due to the complexity of liver metabolism and the immune system, the number of genes that needs to be knocked‐out or knocked‐in remains unknown. Additionally, there is controversy over whether the introduction of humanized genes might cause tissue incompatibility in pigs. 2) Creating pig–primate chimeric embryos by introducing primate or human germline stem cells (GSCs), pluripotent stem cells (PSCs), or induced pluripotent stem cells (iPSCs) into pig blastocysts to produce highly humanized embryos. The advantage of this technique is that it could effectively avoid the impact of unknown xenoantigens. However, the technology problem for increasing the proportion of humanized cells in pig embryos has not yet been completely resolved. Furthermore, chimeric embryos may be involved in complex ethical issues. Abbreviations: HO‐1, Human heme oxygenase‐1; sLSCs, swine liver stem cells; sPSCs, swine pluripotent stem cells; sMSCs, swine mesenchymal stem cells. Adapted with permission from BioRender. Kelai, W. (2024).

By genetically knocking out or editing the GGTA1 gene to create organ transplant donor pigs with GGTA1 gene deletion or inactivation, it is possible to effectively overcome or attenuate the occurrence of HAR after xenotransplantation of porcine livers (Table 1 and **Figure** **3**).^[^ ^19^ ^]^ However, other exposed carbohydrates with α‐galactose residues may still bind to natural antibodies in the recipient's body, potentially triggering rejection reactions. Metabolically enhancing the clearance of α‐galactose residues in donor organs, such as by introducing exogenous α‐galactosidase (GLA) genes, could also effectively control the production of α‐1,3‐galactose and suppress the HAR reactions induced by xenotransplantation.^[^ ^23^ ^]^ α‐Galactosidase is an enzyme that specifically catalyzes the hydrolysis of nonreducing α‐galactose residues at the terminus of α‐galactosides (e.g., galactooligosaccharides, galactoglucomannans).^[^ ^23^ ^]^ The porcine GLA gene shares high homology with the human GLA gene. Introducing the human GLA gene into porcine endothelial cells reduces the surface expression of α‐1,3‐galactose antigens by 78%.^[^ ^23^ ^]^ Additionally, α‐1,2‐fucosyltransferase shares the same substrate specificity as α‐1,3‐galactosyltransferase (*N*‐acetyllactosamine).^[^ ^14^ ^]^ This enzyme could inhibit the formation of Gal antigen epitopes by catalyzing the fucosylation of the common substrate, thereby preventing the galactosylation of the common substrate by α‐1,3‐galactosyltransferase.^[^ ^24^ ^]^ An 80–90% reduction in the synthesis of Gal antigen epitopes was observed in cultured mouse cells transfected with α‐1,2‐fucosyltransferase. α‐2,3‐Sialyltransferase (α2,3ST) and α‐2,6‐sialyltransferase (α2,6ST) are also enzymes that could compete with α‐1,3‐galactosyltransferase for substrates and inhibit the synthesis of Gal antigen epitopes.^[^ ^24^ ^]^

### 4.2 Non‐Gal Carbohydrate Antigens

Complement cascade activation in the context of xenotransplantation has been demonstrated to be influenced not only by Gal antigens but also by non‐Gal antigens, particularly in the realm of carbohydrate metabolism.^[^ ^25^ ^]^ Notable non‐Gal carbohydrate xenogeneic antigens encompass *N*‐acetylneuraminic acid (Neu5Gc) and the Sda/Cad blood group carbohydrates (Table 2).^[^ ^26^, ^27^, ^28^, ^29^ ^]^ Due to genetic mutations arising in the course of evolution, humans lack the enzymatic genes associated with non‐Gal antigens or exhibit significant disparities in gene expression levels compared to pigs. Consequently, the transplantation of only GGTA1 knockout (GTKO) pig livers into humans, does not prevent activation of the complement cascade and humoral immune rejection responses.^[^ ^25^, ^30^ ^]^

#### 4.2.1 *N*‐Glycolylneuraminic Acid (Neu5Gc)

In eukaryotic cells, Neu5Ac is synthesized from uridine 5′‐diphosphate *N*‐acetylglucosamine (UDP‐GlcNAc) in the cytosol. The initial two crucial steps entail the hydrolytic epimerization of UDP‐GlcNAc, leading to the formation of *N*‐acetylmannosamine (ManNAc), followed by phosphorylation to generate ManNAc‐6‐P catalyzed by the bifunctional enzyme UDP‐GlcNAc 2‐epimerase/ManNAc‐6‐kinase (GNE).^[^ ^31^ ^]^ Subsequently, the condensation of phosphoenolpyruvate with ManNAc‐6‐P is facilitated by Neu5Ac 9‐phosphate synthase (NAPS) to produce Neu5Ac‐9‐P. The latter undergoes dephosphorylation by Neu5Ac‐9‐phosphate phosphatase to yield Neu5Ac. Neu5Ac synthesized in the cytosol is translocated to the nucleus, where it combines with cytidine monophosphate (CMP) to form CMPNeu5Ac, the activated form of Neu5Ac, catalyzed by CMP‐sialic acid synthetase.^[^ ^31^ ^]^ The reaction catalyzed by CMAH in the cytosol results in CMP‐Neu5Gc, which is then transferred to the Golgi apparatus (Table 2).^[^ ^31^ ^]^ Various sialyltransferases in the Golgi utilize CMP‐Neu5Gc to form glycoconjugates that are either secreted or expressed on cell surfaces. The key enzyme responsible for Neu5Gc synthesis, CMAH, is genetically inactive in humans, thereby preventing the endogenous synthesis of Neu5Gc‐containing structures.^[^ ^30^ ^]^ A similar loss of Neu5Gc synthesis capability due to independent CMAH inactivation has been observed in New World monkeys.^[^ ^30^ ^]^ However, Neu5Gc‐containing oligosaccharides have been identified in the blood and milk of Old World primates, domesticated animals, pigs, lions, and leopards.^[^ ^32^ ^]^

To date, 22 Neu5Gc derivatives have been documented, encompassing mono‐, di‐, and tri‐O‐acetylation at C4, C5, C7, C8, and/or C9 positions in Neu5Gc.^[^ ^31^ ^]^ Additional modifications include O‐methylation at C5 or C8, O‐acetylation at C9, and O‐sulfation at C8 or C9 of Neu5Gc, with or without O‐acetylation.^[^ ^31^ ^]^ Neu5Gc1,7‐lactone has also been identified. Neu5Gc and its derivatives could form various sialyl linkages with other carbohydrate moieties, including α2‐3 and α2‐6 linkages to galactose, α2‐6 linkages to *N*‐acetylgalactosamine, *N*‐acetylglucosamine, galactose, or glucose, α2‐8 and α2‐9 linkages to another sialic acid molecule, and α2‐5 linkages between polymers of Neu5Gc, thereby adding diversity to sialic acid‐containing compounds.^[^ ^31^, ^33^, ^34^ ^]^ The modification and linkage patterns of sialic acid play pivotal roles in numerous biochemical processes, such as cell signaling, cell–cell interaction, cellular adhesion, inflammation, fertilization, viral infection, malignancies, and the regulation of apoptosis and proliferation.^[^ ^35^, ^36^ ^]^

Despite the inactivation of CMAH in humans, trace amounts of Neu5Gc are found on the cell surface of human tumors and even in normal human tissues.^[^ ^31^ ^]^ The origin of Neu5Gc in human glycoconjugates is likely associated with the consumption of animal‐derived diets, such as red meat and animal milk.^[^ ^31^, ^37^ ^]^ Intriguingly, these internal Neu5Gc compounds do not elicit acute or chronic immune rejection responses. CMAH‐like sequences have been identified in the genomes of some human gut microbiota;^[^ ^38^, ^39^ ^]^ however, the corresponding enzyme activities have not yet been confirmed. In infancy (around 6 months of age), humans develop polyclonal antibodies of IgG, IgM, and IgA types against a diverse array of Neu5Gc‐containing glycans.^[^ ^31^ ^]^ Although the mechanism behind the early formation of anti‐Neu5Gc antibodies remains unclear, it is hypothesized that bacteria colonized in the human body, such as nontypeable Haemophilus influenzae, might incorporate dietary Neu5Gc to form Neu5Gc‐containing epitopes, such as cell surface lipooligosaccharides.^[^ ^31^, ^40^, ^41^, ^42^ ^]^

#### 4.2.2 Sda/Cad Carbohydrates and β1,4GalNAcT‐II

Sda carbohydrate is a high‐frequency epitope present in 90% of Caucasian individuals' red blood cells and is the exclusive surface antigen of the SID blood group.^[^ ^43^ ^]^ The Sda antigen is also detected in adult human tissues, primarily in the digestive tract (colon, kidney, stomach). It is associated with soluble molecules in 96% of Caucasian bodily fluids (urine, milk, meconium, and saliva).^[^ ^44^ ^]^ The structural elucidation of the Sda antigen was achieved in the 1980s, revealing a composition primarily consisting of α2,3‐sialylated lactosamine replacing the β1,4‐linked GalNAc.^[^ ^45^ ^]^ Carbohydrates carrying the Sda epitope exhibit structural diversity, including lacto‐N‐tetraose (Galβ1,3GlcNAc) and lacto‐N‐neotetraose (Galβ1,4GlcNAc) lactosamine chains of types 1 and 2, as well as core 2 and core 3 structures of O‐linked chains.^[^ ^46^, ^47^ ^]^ The biosynthesis of the Sda antigen is driven by a unique gene, the B4GALNT2 gene (encoding β1,4GalNAcT‐II enzyme). β1,4GalNAcT‐II enzyme primarily functions as a Golgi enzyme, serving as a glycosyltransferase that transfers GalNAc from UDP‐GalNAc present in the Golgi to produce the Sda carbohydrate (Table 2).^[^ ^28^ ^]^ The activity of β1,4GalNAcT‐II has been identified in pig intestinum crissum, while the sequence of porcine B4GALNT2 was demonstrated respectively 76% and 70% amino acid identity with human and murine β1,4GalNAcT‐II proteins.^[^ ^28^ ^]^ The cDNA has the same number of exons (11) as human and murine cDNAs.^[^ ^28^ ^]^ Porcine β1,4GalNAcT‐II has a conserved structural motif (SQVTTKYVLWVDDDF), which is also present in murine, sheep, and human β1,4GalNAcT‐II.^[^ ^28^ ^]^ GM2 synthase β1,4GalNAcT‐I is evolutionarily related to β1,4GalNAcT‐II, sharing the same conserved peptide motif.^[^ ^28^, ^48^ ^]^ This motif is believed to contain an acidic divalent cation binding site (DXD) commonly found in glycosyltransferases using UDP‐sugar as the donor substrate.^[^ ^28^ ^]^

Xenotransplantation studies indicate that knockout of β1,4‐*N*‐acetylgalactosaminyltransferase 2 (β1,4GalNAcT‐II) in pigs reduces the binding of human serum antibodies to pig cells.^[^ ^28^, ^29^, ^49^ ^]^ This is believed to be caused by the elimination of GalNAc on the Sda antigen catalyzed by the enzyme β1,4GalNAcT‐II. However, studies on human blood group antigens suggest that only a small percentage (1–2%) of individuals express anti‐Sda antibodies, indicating that the Sda antigen may not be the primary factor causing immune rejection in xenotransplantation.^[^ ^28^ ^]^ Human SID blood types are categorized into Sda+ and Sda− phenotypes based on Sda antigen content, with the Sda+ phenotype further including a Sda++ (or Cad or super‐SID) phenotype characterized by strong reactivity with anti‐Sda antibodies.^[^ ^28^ ^]^ In the human population, the expression level of Sda antigen on red blood cells varies from virtually undetectable to very strong. About 1% of individuals express sufficient Sda antigen to induce massive agglutination by anti‐Sda antibodies. Approximately 80% express enough Sda to cause moderate agglutination.^[^ ^28^ ^]^ All these subjects carry Sda antigens at variable levels on their red blood cells, theoretically not developing anti‐Sda antibodies.^[^ ^28^ ^]^ The remaining 9% show minimal or no agglutination by anti‐Sda antibodies, suggesting that only a tiny percentage of individuals may not express Sda antigens on their red blood cells and possibly develop anti‐Sda antibodies.^[^ ^28^, ^50^ ^]^ Clinical investigations also reveal that individuals with Sda‐negative in blood, over 50%, still have Sda substances in their urine or saliva.^[^ ^51^ ^]^ Only 4% of individuals are Sda‐negative in both blood and urine, and only about half of Sda‐nonsecretors develop anti‐Sda antibodies in their serum, constituting ≈1–2% of the total population.^[^ ^28^ ^]^

Furthermore, porcine endothelial cells are not stained by the anti‐Sda antibody KM694, but they show intense staining by Dolichos Biflorus Agglutinin (DBA).^[^ ^52^ ^]^ This supports that the structural differences of Sda exist on human and porcine red blood cells and/or endothelial cells. The presence of N‐glycolylneuraminic acid (Neu5Gc) in the structure of Sda on pig cells, absent in human cells, might influence the presentation of the Sda epitope and contribute to this difference.^[^ ^52^ ^]^ Expression of porcine β1,4GalNAcT‐II on HEK293 cells increases the binding of both the KM694 monoclonal antibody and the DBA lectin, suggesting that KM694 binding might be influenced by the presentation of the Sda epitope on pig cells, possibly due to the inclusion of Neu5Gc.^[^ ^52^ ^]^ Obviously, the structure of the underlying sugar chains of Sda and Cad antigens in pig cells needs to be elucidated to understand how these structural differences may result in xenoantigens being recognized by human humoral immunity. Previous studies reported an inhibition titer of 32 for Sda substances in pig urine, indicating that even a 32‐fold dilution of pig urine still reduces the strength of agglutination by at least 50%.^[^ ^51^ ^]^ In contrast, the average inhibition titer of Sda+ individuals' urine was 8, and that of newborn infants, children, pregnant, and postpartum patients was 16.^[^ ^51^ ^]^ These data suggest that pig urine has higher Sda activity than human urine, highlighting the need for quantitative and qualitative analysis of molecules containing the Sda/Cad antigen.

In humans, the vWFs are not modified by Sda. Knockout of β1,4 Willebrand T‐II in mice affects the expression pattern of vWFs.^[^ ^53^ ^]^ In addition to inducing the super‐reactivity of porcine Sda antigen, β1,4GalNAcT‐II may also mediate the transfer of GalNAc to vWF.^[^ ^28^, ^53^ ^]^ Studies in Chinese hamster ovary cells demonstrated that transient expression of human vWFs in stable‐expressing β1,4GalNAcT‐II cells was associated with binding of the DBA lectin, while there was no binding of DBA to human β1,4GalNAcT‐II‐negative cells.^[^ ^28^, ^54^ ^]^ This suggests that β1,4GalNAcT‐II could modify the structure of vWFs, potentially contributing to why knockout of B4GALNT2 decreases antibody binding to pig cells. Human natural antibodies could target vWFs on pig endothelial cells. Deficiency of porcine β1,4GalNAcT‐II may affect the glycosylation of vWF and thus decrease antibody binding to the endothelium.^[^ ^55^ ^]^ It has been demonstrated that knockout of B4GALNT2 or vWFs genes significantly extends the survival of pig organs transplanted into primates (Table 1 and Figure 3).^[^ ^56^, ^57^ ^]^

Another hypothesis suggests that the target recognized by natural xenoreactive antibodies in the human body is not the Sda antigen but the β1,4GalNAcT‐II protein encoded by the B4GALNT2 gene.^[^ ^28^ ^]^ This implies that β1,4GalNAcT‐II protein itself may be a non‐Gal antigen. Although there is currently no evidence that β1,4GalNAcT‐II is located on pig cell membranes, studies have reported its expression on the plasma membrane of mouse embryos (blastocyst stage) and in 293T cells as a result of overexpression of a green fluorescent protein‐tagged recombinant protein.^[^ ^28^, ^58^ ^]^ To clarify the role of β1,4GalNAcT‐II in pig xenograft rejection, more experiments are required to explore the distinctions in specific agglutination reactions and antibody‐mediated rejection reactions triggered by B4GALNT2 and Sda.

### 4.3 Protein Antigens

#### 4.3.1 Swine Leukocyte Antigen

The major histocompatibility complex (MHC) in pigs, referred as swine leukocyte antigen (SLA), shares a remarkable structural similarity with human leukocyte antigen (HLA) (Table 2).^[^ ^59^ ^]^ The SLA complex is encoded by genes located on the seventh chromosome of pigs (SSC7), comprising three gene clusters: SLA I, II, and III. SLA I and II regions encode MHC I and II, respectively, while the III region encodes genes crucial for immune defense and inflammation.^[^ ^59^, ^60^ ^]^ The pig MHC I genes are divided into two groups, namely, classical I (Ia) and nonclassical I (Ib) genes.^[^ ^59^, ^60^ ^]^ SLA I includes three functional Ia genes, namely, SLA‐1, ‐2, and ‐3, responsible for presenting peptide fragments derived from intracellular glycoproteins, typically of viral origin, to CD8+ T cells.^[^ ^60^, ^61^ ^]^ The Ib genes, SLA‐6, ‐7, and ‐8, encode splice variants for the formation of different protein isoforms.^[^ ^60^, ^61^ ^]^ Although SLA‐4, ‐5, ‐9, and ‐11 genes are traditionally annotated as pseudogenes due to incomplete coding regions, there are reports of transcriptional translation products for SLA‐9 and SLA‐11.^[^ ^60^, ^62^, ^63^ ^]^ The SLA II region is more compact than the SLA I region and includes MHC II genes involved in antigen presentation, transporter genes related to antigen processing (TAP1, TAP2), and proteasome genes (PSMB8, PSMB9).^[^ ^60^ ^]^ SLA II could be further subdivided into SLA‐DR, ‐DQ, ‐DM, and ‐DO types.^[^ ^60^ ^]^ Unlike the HLA system, SLA II lacks genes encoding DP proteins, resulting in a shorter SLA II gene region compared to HLA II.^[^ ^60^ ^]^ Overall, SLA II genes exhibit strong sequence homology with HLA II genes, and their encoded protein structures share a high degree of similarity in 3D crystal structure.^[^ ^60^ ^]^ In contrast to SLA I and II regions directly involved in MHC gene encoding, the SLA III region encompasses genes crucial for immune defense mechanisms and inflammation. These include genes from the tumor necrosis factor gene family (TNF, LTA, and LTB), steroid 21‐hydroxylase (CYP21A2), complement cascade components (C2, C4A, and CFB), and allograft inflammatory factor 1 (AIF1).^[^ ^60^ ^]^ The region also includes heat shock protein genes (HSP1A, HSP1B, and HSP1L), six different BAT (HLA‐B‐associated transcript homolog) genes, and genes with complex functions such as tenascin‐XB (TNXB) and Notch homolog 4 (NOTCH4).^[^ ^60^ ^]^ The RCCX module gene cluster in the SLA III region differs from the homologous region in HLA III, with four genes (C4B, CYP21A2, TNXA, and STK19P) showing significant interspecies differences in sequence.^[^ ^64^, ^65^ ^]^

Considering the structural similarity between HLA and SLA genes and the >70% sequence identity, HLA‐specific antibodies will likely cross‐react with SLA.^[^ ^60^ ^]^ Mixing human serum with PBMCs from GGTA1/CMAH/B4GALNT2 triple gene knockout (3‐KO) pigs, followed by washing off antiporcine antibodies and then binding to HLA beads, revealed that patients with anti‐HLA‐A antibodies are more likely to cross‐react with class I SLA.^[^ ^56^, ^66^, ^67^, ^68^ ^]^ Similar studies indicate that anti‐HLA class II antibodies may cross‐react with class II SLA.^[^ ^69^ ^]^ Other than being involved in humoral immune rejection, SLA class I and II antigens may be recognized by human CD4+ and CD8+ T cells. Previous studies have reported the lysis of pig cells by human CD8+ T cell clones, with cytotoxicity being inhibited by monoclonal antibodies targeting SLA class I or human CD8.^[^ ^61^, ^70^, ^71^, ^72^ ^]^ These findings suggest that human CD8+ T cells could recognize SLA class I and induce cytotoxic responses. Mixed lymphocyte reactions (MLRs) experiments reveal that human CD4+ T cells exhibit responses to pig cells expressing SLA class II as strong as those against human (allogeneic) cells.^[^ ^61^, ^70^, ^71^ ^]^ Selective inhibition or clearance of CD4+ T cells before xenotransplantation significantly prolongs graft survival compared to the inhibition or clearance of CD8+ T cells.^[^ ^73^ ^]^ Since the majority of immune cells infiltrating pig–baboon or pig–human grafts are CD4+, the immune response against SLA class II is crucial for pig graft survival.

#### 4.3.2 GABARAPL1

GABA type A receptor‐associated protein like 1 (GABARAPL1) is a protein involved in intracellular GABAA receptor transport by binding to microtubule proteins.^[^ ^74^ ^]^ The porcine GABARAPL1‐encoding gene GEC1 shares a high degree of homology with the human GABARAP gene, with a protein similarity of 86%.^[^ ^74^, ^75^ ^]^ Research indicates a close association between GABARAPL1 and the expression of SLA class II antigens (Table 2).^[^ ^76^ ^]^ Knocking out the GABARAPL1 gene in porcine aortic endothelial cells (PAECs) significantly reduces the expression of SLA‐II DR and major histocompatibility complex (MHC) class II transactivator (CIITA).^[^ ^76^ ^]^ Reduced GABARAPL1 in PAECs leads to a substantial decrease in antibody binding in GTKO PAEC‐immunized monkey serum, up to 47%, accompanied by an increase in pig cell survival.^[^ ^76^ ^]^ Immunoprecipitation‐mass spectrometry analysis reveals associations between GABARAPL1 and various transcription factors not extensively studied.^[^ ^76^ ^]^ GABARAPL1 may play a role in regulating the transcription of SLA and non‐Gal antigens through diverse pathways. Previous researches suggest that GABARAPL1 influences the synthesis of various proteins, such as the κ‐opioid receptor, by modulating membrane protein transport and localization efficiency.^[^ ^76^, ^77^ ^]^ While the mechanisms underlying GABARAPL1 mediated regulation in the production of SLA and non‐Gal antigens remain unclear, it is evident that GABARAPL1 is closely linked to immune rejection reactions following xenotransplantation.

#### 4.3.3 COX2

Numerous studies indicate that cyclooxygenase‐2 (COX2) expression is upregulated during allograft rejection, human renal transplantation, inflammation, and cancer (Table 2).^[^ ^78^, ^79^, ^80^ ^]^ With the induction of human serum, COX2 expression in PAECs increases in a time‐ and dose‐dependent mode.^[^ ^80^, ^81^ ^]^ Both COX‐2 mRNA and protein expression are observable at 30 and 60 min post‐treatment, respectively.^[^ ^80^, ^81^ ^]^ Inhibiting COX2 expression reduces PAEC cell death from 20% to 7% after 2 h.^[^ ^80^, ^81^ ^]^ COX2 expression could also be induced by various cytokines, including TNF‐α, IL‐1β, and IL‐17.^[^ ^80^, ^81^ ^]^ Despite these preliminary findings positioning COX2 as a therapeutic target for protecting vascular endothelial cells in xenotransplantation, the exact mechanisms regulating COX2 expression and its function in PAECs remain unknown.^[^ ^80^, ^81^, ^82^ ^]^

#### 4.3.4 Coagulation‐Associated Antigens

Both antibodies mediated and cellular mediated xenograft rejection could cause endothelium injury, accompanied with thrombogenesis and coagulation dysregulation (Table 2 and Figure 2). Damaged endothelium cells expose TF, which bind to activated collagen FVIIa, converting fibrinogen into fibrin and accumulating platelets.^[^ ^83^ ^]^ Interspecific difference of molecular involved in coagulation homeostatic systems enhance this process. The porcine vWFs could bind with the human platelet GP1b to activate platelets to change conformation of human glycoprotein 2b/3a (GP2b/3a) receptors, which bind to fibrinogen to mediate platelet aggregation and endothelial adherence.^[^ ^25^ ^]^ Besides, porcine tissue factor pathway inhibitor and thrombomodulin cannot regulate the activation of human TF and protein C.^[^ ^19^, ^25^, ^84^ ^]^ All of them will benefit the occurrence of xenograft vessel thrombosis. The cell surface antigens are also related to coagulation dysregulation. The difference is that they do not induce thrombogenesis, but cause thrombocytopenia. Porcine Kupffer cells (KCs), the largest macrophage population in liver grafts, could bind with human platelets through CD11b/CD18 receptor to phagocytose them, causing thrombocytopenia and uncontrollable bleeding.^[^ ^25^, ^85^ ^]^ Due to the difference in the amino acid sequence of CD47, pig SIRPα may not be able to recognize human CD47, resulting in Kupffer cell‐mediated phagocytic dysregulation.^[^ ^25^, ^86^ ^]^ Introduction of human coagulation‐associated antigens or cell surface receptors to donor pigs by gene editing, has been shown to be effective in alleviating post‐transplant coagulation dysfunction, but still does not completely prevent such complications.^[^ ^19^ ^]^

### 4.4 New Xenotransplant Rejection Target HMGB1

High Mobility Group Box 1 (HMGB1), interacting with transmembrane receptor TLRs (Toll‐like receptors), is a crucial stimulant for inflammation, cellular damage, and immune rejection. HMGB1 is a DNA‐binding nuclear protein released from endothelial cells, monocytes, macrophages, dendritic cells, and other types of cells through specific secretion pathways or passively during cell death (Table 2). When HMGB binds to TLRs (TLR2, TLR4, and TLR9) on macrophages, it activates NF‐κB and interferon regulatory factor (IRF) pathways, inducing the production of proinflammatory cytokines (TNFα, IL‐1, and IFN‐I) and chemokines.^[^ ^97^ ^]^ Clinical experiments in human allogeneic organs have shown that the presence of HMGB1 is associated with heart, kidney, and lung transplant rejection as well as early liver damage after transplantation.^[^ ^98^, ^99^, ^100^ ^]^ Similarly, HMGB1 plays a vital role in mediating acute xenotransplant rejection. When SD rat hearts are transplanted into adult BALB/c mice, recipient mice experience acute vascular rejection within 6 days of post‐transplantation.^[^ ^97^ ^]^ Along with the occurrence of acute immune rejection, the level of HMGB1 in the plasma post‐transplantation significantly increases in a time‐dependent manner from day 1 to the endpoint.^[^ ^97^ ^]^ Immunohistochemical examination of rejected xenografts shows strong expression of HMGB1 in the cytoplasm of rat cardiomyocytes and infiltrating mouse immune cells.^[^ ^97^ ^]^ Treatment with anti‐HMGB1 antibodies significantly inhibits antibody‐mediated rejection and delays the production of antigraft antibodies after xenotransplantation, extending the survival period of xenogeneic heart transplants from 5 days to 9 days.^[^ ^97^ ^]^ Thus, effectively controlling the production of HMGB1 protein in xenotransplants may be crucial for reducing post‐transplant immune rejection reactions and inducing immune tolerance.

## 5 Immunometabolism of Recipients after Xenotransplantation

The metabolism of the donor pig directly controls the production of xenoantigens, whereas the metabolism of the recipient is closely related to the strength of the immune rejection following xenotransplantation. Pig‐to‐primate liver transplantation will initiate vigorous innate and adaptive immune responses (Figure 2). These immune processes are mediated or regulated by diverse immune cells, such as NK cells, macrophages, neutrophils, dendritic cells (DCs), T cells, and B cells. The metabolic pathways and in generating metabolites have a significant effect on the function of immune cells, affecting several aspects such as development, differentiation, apoptosis, and cell signaling transduction, all of them relevant in the context of xenotransplantation.

### 5.1 Immunometabolism during Immune Rejection

Metabolism is crucial for the maintenance, differentiation, and maturation of immune cells related to xenotransplantation rejection. During acute rejection, there are two main pathways involved in the recognition of xenoantigens: 1) the indirect (donor‐derived antigens are processed and presented by pattern recognition receptors (PRRs) in antigen‐presenting cells (APCs)); 2) the direct (receptors in T cells recognize xenoantigens/donor MHC on donor APCs).^[^ ^101^ ^]^ Damage/danger‐associated molecular patterns recognition during TLR‐driven DC activation is a crucial aspect of transplantation.^[^ ^101^, ^102^, ^103^ ^]^ Several studies have demonstrated that glycogen metabolism supports the initial effector functions of TLR‐activated DCs.^[^ ^101^, ^103^, ^104^ ^]^ Unlike memory T cells and M2 macrophages, which rely on fatty acid oxidation (FAO) to meet their metabolic demands, DCs undergo a metabolic shift from glycogen to glycolysis acquiring a proinflammatory phenotype.^[^ ^105^ ^]^ Glucose blockade promotes a reduction in the expression of costimulatory molecules such as SLA‐I, SLA‐II, CD86, and CD40, which are essential signals in the antigen presentation process during xenotransplantation.^[^ ^60^, ^101^ ^]^

As central players in the process of transplant rejection, T cells are capable of recognizing xenoantigens, activating immune signaling pathways, and coordinating effector responses, leading to organ damage.^[^ ^101^, ^106^ ^]^ The T cells involved in the process of immune rejection primarily consist of helper T cells (Th cells: CD4+), cytotoxic T cells (cytotoxic T lymphocytes: CD8+), and regulatory T cells (Treg cells: CD4+, CD25+, Foxp3+, CD127+).^[^ ^107^ ^]^ These cells work in concert to sequentially accomplish xenoantigen recognition, cellular immune responses, humoral immune activation, immune response regulation, and immunological tolerance. Effector T cells (CD4+ or CD8+) are prominent in rejection reactions after xenoantigen presentation. It has been demonstrated that during acute rejection, the proportion of Th1 and Th17, as well as levels of circulating IFN‐γ and IL‐17, are increased in contrast with reduced levels of Treg cells.^[^ ^108^, ^109^, ^110^ ^]^ Under the guidance of cytokines IFN‐γ and TGF‐β, naïve T cells are activated and differentiated to Th1 and Th17, which display different metabolic patterns with Tregs.^[^ ^101^ ^]^ Th1 and Th17 cells are characterized by a greater reliance on glycolysis and glutaminolysis, resulting in elevated levels of pyruvate, lactate, and intermediates from the pentose phosphate pathway.^[^ ^101^ ^]^ In contrast, Tregs predominantly depend on fatty acid oxidation and oxidative phosphorylation (OXPHOS) to meet their energy requirements.^[^ ^111^ ^]^ Maintained inflammation alters immune cell metabolism, driving a shift toward aerobic glycolysis through signaling pathways involving the phosphoinositide 3‐kinase (PI3K–AKT1–mTOR) axis and upregulation of transcription factors such as MYC and HIF1α.^[^ ^112^ ^]^ This metabolic reprogramming enhances the uptake of amino acids and glutamine, sustaining glutaminolysis and glycolysis, which in turn promotes T cell activation and the production of proinflammatory cytokines.^[^ ^112^ ^]^

Chronic allograft injury is identified by vascular changes, interstitial fibrosis, and organ structures’ atrophy. Fibrosis causes organ damage by a series of cellular and molecular responses to sustained tissue damage.^[^ ^7^, ^19^ ^]^ Tissue inflammation often triggers fibrosis by inducing epithelial‐ and endothelial‐to‐mesenchymal transitions. Some studies described that IL‐33 is released as a response to tissue damage and regulates macrophage differentiation during chronic rejection, preventing disruption of the TCA cycle that shifts macrophage metabolism to anaerobic glycolysis and generates proinflammatory metabolites.^[^ ^113^, ^114^ ^]^ Macrophage stimulation by IL‐33 increases OXPHOS and FA uptake, supporting reparative and regulatory myeloid cell functions.^[^ ^113^, ^114^ ^]^ During chronic rejection, antigens are continually processed by recipient APCs; during this process, it has been described that the metabolic profile governing these late chronic responses involves a skewed response from glycolysis and with a focus on the OXPHOS, which seems to be related to the FAO process.^[^ ^101^ ^]^

### 5.2 Metabolic Pathways Related to Treg Mediated Immune Tolerance

Long‐term acceptance of transplants without immunosuppressive therapy is the ultimate goal in the field of transplantation, and numerous studies are exploring potential therapeutic approaches.^[^ ^106^, ^107^, ^115^, ^116^ ^]^ Reducing or inhibiting the activation of these effector T cells is the key for alleviating or eliminating rejection.^[^ ^107^ ^]^ Treg cells present regulatory or immunosuppressive properties, enabling them to control immune homeostasis by maintaining immunological tolerance to self‐antigens and providing the necessary negative feedback.^[^ ^101^ ^]^ Inducing the activation or proliferation of Treg cell subsets, particularly naturally occurring or thymus‐derived CD4+CD25+Foxp3+ Tregs (nTregs) and adaptive or peripherally induced CD4+ Tregs (iTregs), holds the greatest promise as an alternative or adjunct to reducing the use of existing immunosuppressive agents. The expression of the Foxp3 gene is closely associated with the immunological tolerance induction of Treg cells.^[^ ^117^ ^]^ Both CD4+CD25+Foxp3+ Tregs and adaptive CD4+ Tregs require antigenic activation of host T cell receptors.^[^ ^107^ ^]^ Once activated, Tregs could suppress immune responses at multiple levels, either by directly interacting with effector T cells to inhibit the activation and proliferation of CD4+ and CD8+ T cells, or by indirectly affecting the activity of effector T cells through secreting immunosuppressive cytokines from antigen‐presenting cells.^[^ ^107^ ^]^ Although the process of Treg cells regulating effector T cells to induce immunosuppression is not fully elucidated, there is certainly an association with metabolic and genetic reprogramming of T cells.

Expression of the Foxp3 transcription factor is crucial for the proliferation of CD4+ Treg cells and the maintenance of immune tolerance. The expression of the Foxp3 gene is regulated by IL‐2 expression, T cell receptor (TCR) activation, and mammalian target of rapamycin (mTOR).^[^ ^112^ ^]^ These processes are closely associated with metabolic changes within Treg cells (**Table** **3**). It is increasingly evident that Treg cells exhibit their own metabolic preferences at different activation stages to support their energy and biosynthetic requirements.^[^ ^112^ ^]^ Importantly, some metabolites could also modulate the epigenetics of Treg cells, thereby influencing cell differentiation and function.^[^ ^118^ ^]^ In recent years, extensive research has focused on manipulating these metabolic factors to regulate Treg cell function, aiming to reduce immune rejection reactions following organ transplantation.^[^ ^107^, ^112^, ^119^, ^120^, ^121^, ^122^, ^123^, ^124^, ^125^, ^126^ ^]^ Leveraging cellular metabolism, enhancing Treg cell survival in the inflammatory milieu of transplanted organs, and supporting tissue‐resident or infiltrating Treg cells, may replace conventional immunosuppressive agents to induce immune tolerance effects (Table 3).

**Table 3:** Treg metabolic reprogramming related pathways.

Pathways	Type	Receptors/transporter	Activators	Inhibitors	Activation effect	Inhibition effect	Foxp3 expression (activated pathways)
Fatty acid oxidation (FAO)	Metaboliic	SLC27, SLC27A	SCFAs/AICAR/celastrol	Etomoxir/Emeriamine	Promotes Treg generation/maintains Treg function	Promotes Th1/Th17 generation/decreases Treg suppressive function	Increase
Oxidative phosphorylation (OXPHOS)	Metaboliic	–	SCFAs/glucose/pyruvate/amino acids	Rotenone/Oligomycin/Metformin	Promotes Treg generation/maintains Treg function	Promotes Th1/Th17 generation/decreases Treg suppressive function	Increase
Glycolysis	Metaboliic	GLUT1	Glucose/HIF‐1	2‐DG/D‐mannose/DCA	Promotes Th1/Th17 generation/supports Treg proliferation and migration	Promotes Treg generation/maintains Treg function	Decrease
Krebs cycle (TCA)	Metaboliic	GLUT1	Glucose/dimethyl fumarate	UK5099	Promotes Treg generation/maintains Treg function	Promotes Th1/Th17 generation/decreases Treg suppressive function	Increase
Glutaminolysis	Metaboliic	SLC7A5/SLC1A5/ASCT2	Glutamine/HIF‐1/c‐Myc	BPTES	Promotes Th1/Th17 generation	Promotes Treg generation/maintains Treg function	Decrease
One carbon metabolism	Metaboliic	SLC9A1/SLC1A5/ASCT2/SLC6A14/SLC25A15	Serine/folate	Methotrexate/pemetrexed	Promotes Th1/Th17 generation	Promotes Treg generation/maintains Treg function	Decrease
PI3k/Akt/mTOR signals	Cell signaling	CD278/TCR/IL‐2R/CD28/GLUT1/growth factor receptors	Antigens/growth factors/glucose	Rapamycin/Everolimus/RapaLink‐1	Promotes Treg generation/maintains Treg function	Promotes Th1/Th17 generation/decreases Treg suppressive function	Decrease
IL‐2/STAT5 signals	Cell signaling	IL‐2R	IL‐2	IL‐2 monoclonal antibodies (mJES6‐1/hF5111.2/JES6‐1)	Promotes Treg suppressive function (low‐dose IL‐2)/decreases Treg suppressive function (high‐dose IL‐2)	Enhances Treg expansion/limits cross‐reactivity with human IL‐2 in activated effector T cells	Increase (low‐dose IL‐2)/decrease (high‐dose IL‐2)
TGF‐β/Smads signals	Cell signaling	TGF‐βRI/II	TGF‐β	Pirfenidone/SIS3	Promotes Treg generation/maintains Treg function/promotes Th17 differentiation associated with transplant rejection	Promotes Th1/Th17 generation/decreases Treg suppressive function	Increase
AMPK signals	Cell signaling	IRs/RTKs/LEPR/ARs	Insuliln/Metformin/α‐Adrenergic agonists/Leptin	Doxorubicin/Dorsomorphin	Promotes Th1/Th17 generation (low‐level phosphorylation of AMPK)/promotes Treg generation (high‐level phosphorylation of AMPK)	Enhances inflammatory responses/decreases the efficiency of energy generation	Decrease (low‐level phosphorylation of AMPK) /increase (high‐level phosphorylation of AMPK)
Myc and HIF‐1 signals	Cell signaling	GLUT1	Hypoxia/mTORs/Foxp3	MYCi975/Mycro 3/Chetomin/Chrysin	Impairs iTreg generation and Treg lineage stability (low‐level activation)/promotes Th1/Th17 generation (high‐level activation)	Promotes Treg generation/maintains Treg function	Decrease
Nuclear receptor signals	Cell signaling	PPARγ/LXR/FXR/VDR/RAR	Retinoic acid/SCFAs/Oxysterols/vitamin D3	3‐HB/GW9662/Mifobate/TLC‐2716/vitamin D3 analogues	Promotes Treg generation/maintains Treg function	Promotes Th1/Th17 generation/decreases Treg suppressive function	Increase
CTLA‐4/PD‐1 signals	Cell signaling	TCR/CTLA‐4/PD‐1	Antigens	CTLA‐4 and PD‐1 monoclonal antibodies (Ipilimumab/Tremelimumab/Pembrolizumab/Nivolumab/Pidilizumab)	Promotes Treg generation/maintains Treg function	Promotes Th1/Th17 generation/decreases Treg suppressive function	Increase
HDACs signals	Cell signaling	TCR/c/TLR/IFNGR/IFNAR	Antigens/IFN‐γ/IFN‐α	SAHA/KA1010/TSA/EX‐527	Promotes Treg generation/maintains Treg function	Promotes Th1/Th17 generation/decreases Treg suppressive function	Increase

AMPK: AMP‐activated protein kinase; mTORC: mammalian target of rapamycin; PPAR: peroxisome proliferator‐activated receptors; SCFA: short‐chain fatty acids; SLC: solute carrier proteins; GLUT: glucose transporters.

During proliferation and differentiation, Treg cells heavily rely on OXPHOS to generate adenosine triphosphate (ATP) to meet their energy demands.^[^ ^127^ ^]^ Acetate and fatty acids serve as the primary energy sources for Treg cells.^[^ ^127^ ^]^ In the early stages of naive T cell differentiation, the regulation of the glycolysis pathway could influence the Th17/Treg balance.^[^ ^127^ ^]^ Activation of the glycolysis pathway could induce differentiation of naive T cells into Th1, Th2, or Th17 cells, suppress Foxp3 gene expression, and limit Treg cell production. Glycolysis is a relatively inefficient pathway for cellular ATP production (only two molecules), which is disadvantageous for Treg cell survival in hypoxic, acidic environments. However, this does not imply complete abandonment of the glycolysis pathway by Treg cells. Studies have found occurrences of the glycolysis pathway in proliferating, migrating, and effector Treg cells.^[^ ^127^, ^128^, ^129^ ^]^ Active glycolytic metabolism activates the mTORC1 pathway, enhancing cellular glucose uptake.^[^ ^130^ ^]^ High glucose environments weaken the immunosuppressive function of Treg cells.^[^ ^130^ ^]^ To avoid this adverse effect, Treg cells accelerate the synthesis of lactate and phosphoenolpyruvate during proliferation and migration, increasing the adaptability of Treg cells to their environment.^[^ ^130^ ^]^

OXPHOS metabolic reprogramming driven by FAO maintains the suppressive phenotype of Treg cells, further promoted by the expression of Foxp3.^[^ ^131^ ^]^ Mature Treg cells highly express the fatty acid transporter CD36 and activate the PPAR‐b pathway to uptake exogenous fatty acids for survival and suppressive function maintenance.^[^ ^132^ ^]^ Simultaneously, Treg cells actively remodel the transcription factor SREBP‐dependent de novo lipid biosynthesis, aiding in TCR‐induced functional maturation and the expression of PD‐1 checkpoint molecules.^[^ ^133^ ^]^ The AIM inflammasome could regulate Akt phosphorylation, mTOR, and Myc signaling, further influencing lipid‐mediated OXPHOS processes within Treg cells.^[^ ^134^ ^]^ Similar to lactate, Treg cells could utilize exogenous oleic acid to counteract the negative effects of high glucose uptake, enhancing FAO‐driven OXPHOS metabolism, forming a positive feedback mechanism, increasing Foxp3 expression, and STAT5 phosphorylation, thereby maintaining immune suppression.^[^ ^135^ ^]^

Amino acid metabolism supports the synthesis of functional proteins and nucleotides during the rapid growth phase of Treg cells.^[^ ^136^ ^]^ During the proliferation and activation of Treg cells, subunits of amino acid transporters, including SLC7A5, SLC43A2, SLC7A1, particularly SLC3A2 and SLC7A11, are upregulated.^[^ ^136^, ^137^, ^138^, ^139^ ^]^ These proteins control the uptake of essential extracellular amino acids and exchange intracellular and extracellular amino acids in Treg cells.^[^ ^136^ ^]^ Studies have shown that reducing the intake of essential amino acids such as isoleucine, leucine, and valine decreases the proliferation and suppressive capacity of mouse Treg cells.^[^ ^136^, ^137^, ^138^, ^139^ ^]^ Amino acid transporters regulate the metabolic stability of Treg cells by enhancing amino acid uptake while modulating the mTORC1 signaling pathway.^[^ ^137^ ^]^ Additionally, amino acid metabolic enzymes and intermediates are crucial determinants of Treg cell induction. For example, the activity of the amino acid consumption enzyme indoleamine 2,3‐dioxygenase (IDO) could strongly promote in vitro Treg cell differentiation.^[^ ^140^, ^141^ ^]^ Tryptophan metabolites, particularly quinolinic acid generated by IDO, could bind to aromatic hydrocarbon receptors on T cells, promoting Treg cell proliferation.^[^ ^140^, ^141^ ^]^

In Treg cells, glucose, lipid, and amino acid metabolisms are closely related to the mTOR signaling pathway. The mTOR pathway serves as a switch for T cell glycolysis, determining the differentiation of naive T cells, and affecting the Th17/Treg balance in the blood and tissues.^[^ ^142^ ^]^ The mTOR complex consists of mTORC1 and mTORC2, with mTORC1 playing a primary role in regulating T cell metabolism.^[^ ^143^ ^]^ Phosphorylation signals upstream of mTOR are initiated by stimulation of the TCR complex or CD28. This triggers sequential phosphorylation of phosphatidylinositol 3‐kinase (PI3K), phosphoinositide‐dependent kinase 1 (PDK1), and protein kinase B (Akt).^[^ ^143^ ^]^ Akt subsequently inhibits the tuberous sclerosis complex (TSC1/2) to maintain the activity of Ras homolog protein (Rheb).^[^ ^143^ ^]^ Ultimately, Rheb could directly and indirectly increase the activation of mTORC1.^[^ ^143^ ^]^ Activation of mTORC1 inhibits the expression of Foxp3, restricts the immune suppressive function of Treg cells, induces autoimmunity, and disrupts tissue immune homeostasis.^[^ ^144^ ^]^ Therefore, rapamycin blockade of the mTOR signaling pathway could effectively enhance immune tolerance in organisms.^[^ ^144^ ^]^ Signals associated with Foxp3 expression, such as IL‐2, could also regulate Treg cell metabolism through the PI3K–AKT–mTOR signaling pathway.^[^ ^145^ ^]^ Once binding to the IL‐2 receptor IL‐2R, IL‐2 activates the JAK‐STAT pathway, indirectly controlling the activation of mTORC1 and hypoxia‐inducible factor 1 transcription complex (HIF1α/HIF1β), thereby regulating the uptake of glucose and other nutrients and the occurrence of glycolysis in Treg cells.^[^ ^145^ ^]^ Additionally, IL‐2 maintains the expression of the amino acid transporter SLC7A5, transporting essential amino acids such as leucine into the cell for critical protein synthesis, such as CTLA4.^[^ ^145^ ^]^

### 5.3 Immunosuppression by Immunometabolism Reprogramming

Compared to allogeneic organ transplantation, xenotransplantation is more prone to immune rejection reactions, namely, graft‐versus‐host disease (GVHD). As a complex metabolic organ in mammals, the liver presents a higher difficulty in xenotransplantation. Despite the improved tissue compatibility of pig livers after gene knockout or gene editing, there are still many unknown hepatic metabolites that may trigger post‐transplant immune rejection (Table 1 and Figure 3).^[^ ^19^, ^146^ ^]^ Inhibition of glycolysis, either by targeting mTORC1 or downstream HIF1α signaling, facilitates a shift from Th1 and Th17 to Treg differentiation.^[^ ^101^ ^]^ Transplant recipients are administered therapeutic doses of immunosuppressive agents to preserve the integrity of tissues and organs and to prevent immune rejection. These pharmacological agents significantly influence the metabolic behaviors of immune cells. Calcineurin inhibitors, such as cyclosporine and pimecrolimus, represent the primary immunosuppressive regimens, affect cellular enzymatic activity (mediated by cytochrome P450), and regulate IL‐2 production by immune cells.^[^ ^101^ ^]^ Corticosteroids could cause immunosuppression mainly by blocking the expression of proinflammatory cytokines and sequestration of CD4+ T‐lymphocytes in the reticuloendothelial system.^[^ ^101^ ^]^ Another important group of immunosuppressant regiments, mTOR inhibitors, such as rapamycin, through glycolytic metabolic reprogramming, impede effector T cell proliferation by blocking growth factor‐mediated T‐cell expansion in response to IL‐2 while also disrupting the metabolic shift for maintaining Th17/Treg balance.^[^ ^101^ ^]^ However, currently used immunosuppressive regimens to control graft immune responses are not ideal solutions because they are highly nonspecific, require lifelong use, and predispose transplant patients to opportunistic infections and tumors, some of which may be fatal.^[^ ^147^ ^]^ Common broad‐spectrum immunosuppressive drugs, including corticosteroids, calcineurin inhibitors, and rapamycin‐targeted immunosuppressive drugs, readily induce immunocompromise in patients, leading to postoperative infections.^[^ ^123^, ^148^ ^]^ The mortality rate from organ failure and pathogen infections in transplant recipients using steroid immunosuppressive agents could even reach 90%.^[^ ^149^ ^]^ Traditional immunosuppressive agents exhibit good efficacy against hyperacute and acute rejection shortly after surgery but fail to eliminate long‐term chronic immune rejection reactions and may induce thrombosis and coagulation disorders (Figure 1).^[^ ^19^ ^]^ To improve the safety and controllability of inducing immune tolerance, novel low‐toxicity mTOR inhibitors, such as everolimus and RapaLink‐1, and advanced strategies for inducing Treg and tolerogenic APCs, have been developed.^[^ ^150^, ^151^ ^]^ The details of these methods will be introduced in Section 6.

### 5.4 Metabolic Barriers after Liver Xenotransplantation

#### 5.4.1 Incompatibility of Glucose, Lipid, and Amino Acid Metabolism

The liver is both an important metabolism and immune organ. After liver xenotransplantation, immune cells in transplant recipients are regulated by metabolites from donor pig liver. The physiologic differences of livers between human and pig, may cause the problems of metabolic incompatibility, which potentially destroy the immune system of transplant recipients and induce severe complications, such as metabolic disorders and tumors.^[^ ^19^ ^]^ It has been demonstrated that the qualitative function of enzymes that regulate glucose, lipid, and protein metabolism does not appear to differ markedly from those in humans.^[^ ^101^ ^]^ However, humans, in contrast to pigs, exhibit some extrahepatic gluconeogenesis. During the anhepatic phase of liver transplantation, blood glucose in humans showed no significant changes, and blood lactate only increased slightly. In contrast, blood glucose in anhepatic pigs decreased by half within 26 min, while blood lactate continued to rise.^[^ ^101^, ^152^ ^]^ Moreover, the distribution of porcine glucose transporter proteins (GLUTs) in the liver, intestine, and muscle differs from that of humans.^[^ ^101^, ^153^ ^]^ It indicates that there may be different regulation systems of glucose metabolism between humans and pigs. Compared to humans, pigs rely more on the liver for blood glucose regulation, and there are differences in their sensitivity to glucose hormones. The insulin dose required to stabilize blood glucose is significantly lower in pigs than in macaques and humans of similar body weights. Pigs differ from macaques and humans by a much lower C‐peptide level in blood and a two‐ to sevenfold lower C‐peptide response to arginine stimulation. Interestingly, during the intravenous glucose tolerance test, glucose injection led to a rapid threefold decrease in plasma glucagon within 2 to 10 min, while a significant increase in plasma insulin took 20 min to occur.^[^ ^154^, ^155^ ^]^ This suggests that pigs rely more on glucagon than insulin for blood glucose regulation. Insulin and glucagon directly regulate the pathways that control glucose entry (GLUT1, GLUT6, etc.) and biochemical enzymes (fructose‐2, 6‐bisphosphate, fructose‐6‐phosphate, etc.) involved in glycolysis and glycogenesis, which are crucial for T, cell maintenance, and differentiation.^[^ ^156^ ^]^ Therefore, the ability of the transplanted pig livers to cooperate with the original human islets in regulating blood glucose may influence the long‐term immune function and incidence of immune rejection in post‐transplant recipients. In addition, whether the transplanted porcine liver could synergize with other tissues, such as the intestines, for blood glucose regulation is still unknown. In rats, similar with humans, leptin could also suppress the production of glucagon and corticosterone, increase glucose uptake, and inhibit hepatic glucose output.^[^ ^157^ ^]^ It has been described that in rat hepatocytes, exposure to leptin secreted by the intestine (3 h, 50 and 100 nm) attenuated glucagon stimulated hepatic gluconeogenesis by 35% and 38%, respectively.^[^ ^158^ ^]^ However, leptin does not produce any significant acute effects in porcine hepatocytes. Leptin decreases glucagon induced PEPCK mRNA levels by 25% to 35% in rat hepatocytes, but not in porcine hepatocytes.^[^ ^158^ ^]^ It indicates leptin acts differentially between species and interferes with the metabolic stability of xenograft recipients.

In the pig‐to‐primate liver xenotransplantation model, amino acids and lipids profiles of serum and plasma samples from pre‐transplant pigs and post‐transplant baboons were analyzed. Compared to both porcine and baboon baseline levels, the amino acids and lipids profiles of recipients remain stable after xenotransplantation.^[^ ^159^ ^]^ However, due to the ischemia/reperfusion injury, there is a threefold increase in l‐ornithine and more than a tenfold decrease in l‐arginine post‐transplantation.^[^ ^160^ ^]^ Ischemia/reperfusion injury is linked to mitochondrial damage associated with inhibition of ATPases and nitric oxide (NO) signaling related to cell apoptosis.^[^ ^161^ ^]^ These damages are unavoidable consequences after xenotransplantation and may aggravate the metabolic incompatibility of liver xenografts. It has been demonstrated that ischemia/reperfusion injury could induce lipid metabolic reprogramming in pig liver by activating ALOX12–12‐HETE–GPR31 signaling axis.^[^ ^162^, ^163^ ^]^ The crucial enzyme arachidonate 12‐lipoxygenase (ALOX12) in the arachidonic acid metabolic pathway and its product 12‐hydroxyeicosa tetraenoic acid (12‐HETE) are upregulated during the early stages of hepatic ischemia/reperfusion injury.^[^ ^162^, ^163^ ^]^ This lipid reprogramming promotes liver dysfunction, cell death, and inflammation induced by hepatic ischemia/reperfusion injury.^[^ ^162^, ^163^ ^]^ Blocking 12‐HETE production by ALOX12‐specific inhibitors may alleviate ischemia/reperfusion injury, and the resulting metabolic incompatibility.^[^ ^162^, ^163^ ^]^ However, its long‐term effects still require further evaluation, and it is not clear the upstream signals that lead to the reprogramming of lipid metabolism during ischemia.

In addition to lipid metabolic incompatibility due to ischemia/reperfusion injury, there are inherent interspecies differences in lipid metabolism between pigs and humans. Cholesterol is an essential element for both humans and pigs to construct the cell membrane and synthase steroid hormones. However, it has been described that the serum concentration of cholesterol in pigs (80 ± 9.3 mg dl^−1^) is much lower than in humans (≈160 mg dl^−1^).^[^ ^164^ ^]^ The relative hypercholesterolemia of humans may be detrimental to pig liver xenografts, contributing to the early development of graft vasculopathy.^[^ ^164^ ^]^ Furthermore, the molecular structure of lipoproteins, such as high‐density, low‐density (LDL), and very low‐density lipoproteins, is species‐specific and differs by ≈40% between pigs and humans.^[^ ^164^ ^]^ After liver xenotransplantation, the structure of the circulating lipoproteins in the recipients will diverge from those normally present. Due to a greater number of apo‐B‐receptors in the porcine liver, compared with humans, there is a remarkably lower binding capacity of LDL with cholesterol in pigs.^[^ ^164^ ^]^ Therefore, compatibility between cholesterol and lipoproteins will not be guaranteed after liver xenotransplantation, since the former is of human origin and the latter retains porcine characteristics. Theoretically, unstable cholesterol levels in the blood could induce changes in bile, bile salts, and bilirubin metabolism. However, clinical studies have shown that no abnormalities in bile secretion have been detected in the short term after human patients received liver xenografts.^[^ ^20^ ^]^ Currently, patients, received liver xenografts, are either brain‐dead or in the end stages of liver failure, making it impossible to observe long‐term metabolic changes after transplantation.^[^ ^19^, ^20^ ^]^ It is still unclear that in the long‐term (years later), whether human recipients will face metabolic disorders due to cross‐species differences. For cardiac and renal xenotransplantation, multiomics approaches, including metabolomics, proteomics, and transcriptomics, have been utilized to accurately analyze metabolic incompatibility in recipients.^[^ ^160^, ^165^, ^166^ ^]^ However, few reports have been found from similar studies on liver transplantation.

#### 5.4.2 Differences in CYP‐Mediated Metabolism of Drugs

The liver serves as the primary site for metabolizing xenobiotics, steroids, cholesterol, and bile acids. During the post‐transplantation stage, a large amount of immunosuppressive and xenobiotics is injected into the recipient's body. However, there is still no clinical report to prove that pig liver could fully metabolize human medications. The unknown metabolites produced by porcine liver potentially result in the metabolic syndromes or graft failure. In the liver, cytochrome P450 (CYP450)‐mediated metabolism constitutes a crucial initial step in the oxidation of numerous known xenobiotics, including drugs (Figure 2).^[^ ^87^ ^]^ The CYP450 complex encompasses various subfamilies such as CYP1A, CYP2A, CYP3A, CYP2B, CYP2C, CYP2D, and CYP2E, each with overlapping but distinct substrate specificities, different inhibitors and inducers.^[^ ^88^ ^]^ The total quantity of hepatic CYP450 enzymes is similar between pigs and humans (365 vs 240–303 pmol mg^−1^ protein). However, relative levels of CYP450 enzyme subfamilies differ, with pigs exhibiting lower proportions of CYP2C, CYP1A2, and CYP3A (10 vs ≈25%, 4 vs 10–18%, and 14 vs 32–38% in pigs vs humans, respectively) and higher proportions of CYP2A, CYP2B, CYP2D, and CYP2E (31 vs ∼7%, 31 vs ≈1%, 25 vs ∼2%, and 13% vs 8–17% in pigs vs humans, respectively).^[^ ^89^, ^90^, ^91^, ^92^ ^]^

In humans, the CYP1 family metabolizes ≈13% of substances, including carcinogens, estrogens, mycotoxins, bilirubin, and a small amount of antidepressants or analgesics.^[^ ^88^, ^93^ ^]^ This cytochrome family is influenced by the same inducers across different species. In miniature pigs and humans, CYP1A activity is gender‐related but manifests differently. In minipigs, females exhibit two to four times higher activity than males. While in humans, the Caucasian males display two to four times higher activity than females.^[^ ^93^ ^]^

The CYP2 family metabolizes 50% of all prescription drugs, including nicotine, nitrosamines, and aflatoxin.^[^ ^88^, ^93^ ^]^ In pigs, porcine CYP2A19 constitutes 34% of hepatic CYP, sharing 90.1% DNA sequence homology with human CYP2A13 and 87.2% homology with human CYP2A6.^[^ ^94^ ^]^ CYP2A19 activity in female pigs is 70 times higher than in males, but upon castration, male animals experience a tenfold increase in CYP2A19 activity.^[^ ^88^ ^]^ Unlike pigs, human CYP2A19 protein activity shows no gender differences, possibly affected by intermediary hormones.^[^ ^93^ ^]^ CYP2B enzymes are scarce in both pig and human tissues but are involved in the metabolism of significant drugs such as diazepam, lidocaine, cyclophosphamide, and tamoxifen.^[^ ^88^ ^]^ Human CYP2B6 shares 81.1% DNA sequence homology with porcine CYP2B22.^[^ ^95^ ^]^ Porcine CYP2B expression decreases with age, but no gender differences.^[^ ^96^ ^]^ In humans, CYP2C metabolizes 25% of drugs, including clozapine, propofol, estrogens, testosterone, and methadone.^[^ ^93^ ^]^ Porcine CYP2C33v4 exhibits 62.6% homology with human CYP2C9.^[^ ^94^ ^]^ While porcine CYP2C enzymes show some cross‐reactivity with substrates tested in human studies, their metabolism of human CYP2C‐specific substrates is almost negligible.^[^ ^93^ ^]^ CYP2D metabolizes antidepressants, antipsychotics, and beta‐blockers in humans. Due to polymorphism, CYP2D exhibits high interindividual variability in humans.^[^ ^93^ ^]^ Porcine CYP2D25 constitutes 25% of hepatic CYP, sharing 77.6% DNA sequence homology with human CYP2D6.^[^ ^94^ ^]^ Porcine CYP2D has not been fully explored. Its potential substrates have been shown to be metabolized by porcine CYP2B.^[^ ^88^ ^]^ CYP2E may be involved in the formation of highly activated toxic or carcinogenic metabolites.^[^ ^88^ ^]^ There is 75% sequence homology of CYP2E between human and porcine.^[^ ^88^ ^]^ Alcohol, high‐fat diets and stress could activate the CYP2E‐mediated metabolism and regulate the processes of gluconeogenesis, hepatic cirrhosis, diabetes, and cancer.^[^ ^88^, ^94^ ^]^

The CYP3 family is an important drug‐metabolizing family in humans, responsible for the metabolism of ≈34% of compounds, constituting 30% to 40% of total hepatic CYPs.^[^ ^93^ ^]^ In addition to drugs, this enzyme could also metabolize sex hormones and polycyclic aromatic.^[^ ^88^ ^]^ The reactive substrates of CYP3A are similar between humans and pigs.^[^ ^88^ ^]^ In porcine, CYP3A expression varies with age and breed. Yucatan pigs demonstrate higher CYP3A activity compared to Göttingen miniature pigs and traditional breeds.^[^ ^93^ ^]^ Porcine CYP3A primarily presents in the liver, intestines, and kidneys. Bama miniature piglets express higher levels of CYP3A29 in the duodenum than in the liver, while the opposite is observed in adult pigs.^[^ ^93^ ^]^

There are broad similarities between humans and pigs in cyp450‐mediated drug metabolism, but undeniable interspecies differences remain. These differences are evident in drug metabolism pathways and enzyme activity. Therefore, special attention needs to be paid to drug dosage and contraindications after xenotransplantation. The isoforms CYP3A4 and CYP3A29 of porcine metabolize a large number of commonly prescribed immunosuppressants and anticoagulants used in xenotransplantation, such as cyclosporine, tacrolimus, corticosteroids, statins, and warfarin.^[^ ^164^ ^]^ Although few studies have explored the difference between pigs and humans in CYP3A mediated metabolism of immunosuppressants, it has been described that immunosuppressants with equivalent dosages to humans could lead to overimmunosuppression, post‐transplant lymphoproliferative disease and graft failure in pigs receiving allogeneic liver transplantation.^[^ ^167^, ^168^, ^169^ ^]^ In addition, there are cross‐species differences in CYP2C and CYP2D mediated metabolism of anti‐inflammatory and immunomodulatory drugs. Compared with humans, the metabolism of diclofenac (substrates of CYP2C9) is deficient in pig liver microsomes and does not show corresponding activity.^[^ ^167^ ^]^ In contrast, dextromethorphan (substrates of CYP2D6) exhibited a very extensive and rapid metabolism in pig liver microsomes.^[^ ^167^ ^]^ Therefore, humans transplanted with pig livers may no longer be suitable for these anti‐inflammatory or immunomodulatory agents with low metabolic efficiency. Similar drugs, such as bufalin and schizandrin, which have in vitro antitumor effects against various malignant cell lines, have shown species differences in metabolism pathways.^[^ ^170^, ^171^ ^]^ It has been reported de novo malignancies following liver transplantation.^[^ ^172^ ^]^ When xenotransplant recipients experience similar complications, the use of such immunomodulators or anticancer drugs may lead to various toxicological effects.

### 5.5 Incompatibility of Immune Cells across Species

The liver is home to a multitude of immune cell types, including Kupffer cells, NK cells, γδ T cells, etc. These immune cells regulate innate and adaptive immune responses as well as the liver's immunological tolerance by promoting DC cell maturation, secreting chemokines and cytokines to activate neutrophils, T cells, and others (Figure 2).^[^ ^173^ ^]^ Distinguishing the transcriptomic characteristics and functional differences between human and pig immune cells is crucial for improving survival after pig liver xenotransplantation. Single‐cell transcriptomics analyses of immune cells from humans and pigs show that the subtypes of immune cells are broadly consistent between the two, but there are numerous differences in cell numbers and key marker gene expression patterns, which also contribute to their functional differences. The diversity and immune response capabilities of T cell receptors (TCRs) in different species lead to different T cell lineage differentiation; CD2^‐^γδ T exists only in pigs and not in humans;^[^ ^174^ ^]^ pigs' CD2^‐^CD8^+^γδ T cells have high functional homology with humans' CD2^‐^CD4^+^γδ T cells, but CD4 T cells are more active in humans, while CD8 T cells are usually more abundant in pigs, resulting in significant differences in the immune regulatory effects of T cells between species.^[^ ^175^, ^176^ ^]^ NK cells are important innate immune cells, and the transcriptomic features of NK cells residing in the livers of humans and pigs are very similar; pig liver‐resident NK cells mainly express genes related to adaptive immunity.^[^ ^177^ ^]^ After organ xenotransplantation, host NK cells initiate and regulate immune rejection responses, recruiting to the donor organ site. The molecular incompatibility between human and pig CD31^[^ ^178^ ^]^ causes pig endothelial cells to activate and secrete chemokines like CXCL8 acting on human neutrophils through CXCR2 and PAF receptor, producing proinflammatory chemokines and cytokines, activating human NK cells, ultimately leading to endothelial cell damage.^[^ ^179^ ^]^

KCs express the CD11b/CD18 receptor (also named macrophage antigen complex 1, Mac1, and αmβ2 integrin) and account for ≈90% of all resident macrophages in the liver.^[^ ^25^ ^]^ Pig KC could bind with human CD18, and the species difference in macrophage antigen complex‐1 in KC mediates recognition action, causing thrombocytopenia after liver xenotransplantation.^[^ ^85^, ^180^ ^]^ Macrophages, including KCs, have species‐specific CD47 signaling regulatory protein SIRPα recognition mechanisms, regulating the phagocytic action of macrophages.^[^ ^86^ ^]^ However, the amino acid sequence similarity between human and pig CD47 is only 73%.^[^ ^86^ ^]^ After liver xenotransplantation, pig SIRPα may not be able to recognize human CD47, resulting in phagocytosis of human platelets.^[^ ^25^, ^86^, ^185^ ^]^ While the endothelial cells of liver blood vessels with pig CD47 expression will not be recognized by human SIRPα‐expressing macrophages, which will activate the execration of inflammatory cytokines and produce xenograft injury.^[^ ^25^, ^86^ ^]^ However, it has also been suggested that KCs may protect liver grafts. KCs phagocytose apoptotic cells in liver graft and secrete inhibitory factors, such as TGF‐β, IL‐10, and PGE‐2, resulting in the creation of a local immunosuppressive microenvironment that reduces lymphocyte function, reducing acute rejection and improving liver function.^[^ ^181^, ^182^ ^]^ KCs overexpress the F1 scavenger receptor (SCARF1) to stimulate the calcium‐dependent PI3K‐AKTSTAT3 signaling pathway, enhancing their phagocytosis to eliminate the released immune antigens (CRT, ERp57, and GP96) from dead cells to promote immune tolerance post transplantation.^[^ ^181^, ^183^ ^]^ Besides, KCs have remarkable plasticity that they could use PRRs and TLRs to detect exogenous or endogenous signals within different microenvironments for displaying distinct cellular phenotypes, namely, M1 and M2 type.^[^ ^181^ ^]^ Polyunsaturated fatty acids, the degradation product of apoptotic cells, facilitate metabolic reprogramming of KCs through the activation of the PPARγ signaling pathway, which promotes the polarity of KCs to M2 type.^[^ ^181^, ^184^ ^]^ Stimulated M2 phenotype increases the expression of anti‐inflammatory mediators, including IL‐10, SCARF1, mannose receptors, and tissue remodeling molecules like TGF‐β, thereby reducing acute rejection after liver transplantation.^[^ ^181^, ^184^ ^]^ Therefore, exploring the methods to change the polarity of KCs to M2 type may be of great significance to induce immune tolerance after liver xenotransplantation.

## 6 Strategies to Overcome Liver Xenograft Destruction

Current strategies to overcome liver xenograft destruction caused by immune rejection could be classified into two categories: 1) pre‐transplantation techniques of targeting donor antigens, such as donor gene modification, humanized chimeric embryos; 2) post‐transplantation techniques of inducing immune tolerance in xenotransplantation recipients. We will introduce their progress and development directions in the following sections.

### 6.1 Pre‐Transplantation Techniques (Donor Specific)

Donor pig livers produced with gene modification have already been successfully transplanted into patients with liver failure. Knocking‐out Gal and non‐Gal antigens related genes, such as GGTA1, CMAH, and B4GALNT2, could significantly prevent the occurrence of HAR, and reduce the incidence of DXR. However, it does not mean that the clinical application of liver xenotransplantation is absolutely safe and reliable. The potential existence of unknown xenoantigens and the metabolic risk after gene knockout are still unknown. Improvements in pre‐transplantation techniques to produce more reliable xenografts are necessary.

#### 6.1.1 Modification of Homologous Genes

Gene knockout strategy is applicable for eliminating porcine‐specific antigen genes, such as GGTA1, CMAH, and B4GALNT2. However, the question of which strategy—knock‐out, knock‐in, or replacement—is more effective for homologous genes shared between pigs and humans remains challenging. The MHC or SLA antigens in pigs share a remarkable structural similarity with human HLA antigens, and more than 70% sequence identity.^[^ ^59^, ^60^ ^]^ SLA gene family is highly diverse and comprises many large‐segment genes.^[^ ^59^, ^60^ ^]^ Completely knocking‐out SLA class I or SLA class II genes is challenging and likely to disrupt the pig's immune system, leading to developmental disorders.^[^ ^61^, ^186^ ^]^ Instead, researchers tried to target the β‐2‐microglobulin (β2m) gene, which paired with all SLA class I heavy chains. It has been reported that β2m knockout pigs show reduced levels of CD8+ T cells in peripheral blood, and a decreased level of human IgG and IgM in in vitro xenoreactive reactions.^[^ ^61^, ^186^, ^187^ ^]^ Regarding SLA class II antigens, introducing a human dominant‐negative mutant class II transactivator (hCIITA‐DN) gene into the donor pigs could significantly decrease human CD4+ T‐cell proliferation.^[^ ^188^ ^]^ In vitro testing, compared to wild‐type porcine aortic endothelial cells (pAECs), pAECs with hCIITA‐DN gene significantly reduced the expression of SLA II antigens, suppressed the human CD4+ T‐cell response by 40–50%, and inhibited the activation of pAECs.^[^ ^188^ ^]^ However, simply knocking out β2m or introducing hCIITA‐DN does not seem to eliminate the influence of SLA antigens completely. It has been reported that transplantation of β2m(−)/CIITA(−) or hCIITA(+) pig xenografts could still trigger DXR and AMR.^[^ ^187^, ^188^ ^]^ Alternative strategies to the complete knock‐out of the β2m or knock‐in hCIITA would be CRISPR/Cas9‐mediated mutation that results in low SLA phenotypes or replacement of SLA genes with homologous HLA genes.^[^ ^61^, ^189^ ^]^ Theoretically, these two approaches could prevent an immune response in pig‐to‐human or nonhuman primate (NHP) xenotransplantation more effectively. However, their technical implementation is highly challenging. At present, it has not been reported that the successful replacement of pig SLA genes with their homologous human genes. Editing SLA genes may lead to complex immune metabolic reprogramming.

#### 6.1.2 Humanized Chimeric Organs

Biological incompatibility between pigs and NHPs/humans due to the complexity of liver metabolism and the immune system, significantly shorten the lifespan of liver xenografts.^[^ ^19^ ^]^ Even now the precise number of genes that need to be knocked‐out or knocked‐in remains unclear. Humanized chimeric organs created through interspecies blastocyst complementation or gene manipulation in severe combined immunodeficiency (SCID) pigs are a promising alternative to overcome this barrier. Previous researchers have successfully implanted human induced pluripotent stem cells (hiPSCs) into pig embryos and generated 3D, functional, and transplantable organs (such as liver bud, mesonephros, skeletal muscle, etc.) within pig fetuses from the 4CL culture condition in vitro.^[^ ^190^, ^191^, ^192^, ^193^ ^]^ Impressively, they yielded chimeric fetuses by embryonic day 28, and 40–60% of cells in these solid human‐like organs are derived from hiPSCs.^[^ ^190^, ^191^ ^]^ Despite these exciting progresses, there remain challenges in ethical concerns and virus infection risks for both animals and humans.^[^ ^190^ ^]^ To overcome these barriers, researchers have tried to transplant human hepatocytes into live piglets to construct transplantable chimeric organs. Piglets with the depletion of genes of recombination activating 2 (RAG2), interleukin 2 receptor subunit gamma (IL2RG), and fumaryl acetoacetate hydrolase (FAH), would lack mature T, B, and NK cells.^[^ ^19^, ^194^ ^]^ SCID pigs with RAG2−/−IL2Rg−/−FAH−/− have been demonstrated the potential ability to be engrafted with a large number of human hepatocytes without immune rejection.^[^ ^19^, ^194^ ^]^ Despite the fact that this technology has not yet been tested in clinical trials, its potential could not be overlooked. We believe that liver chimera from genetically engineered pigs will one day successfully replace human grafts to be transplanted into patients.

#### 6.1.3 Machine Perfusion and Partial Reprogramming

Ex vivo preservation of organs is crucial for successful xenotransplantation. In the past decade, technologic advances brought renewed efforts to develop normothermic machine perfusion (NMP) devices. Instead of static cold storage, the NMP method has lots of advantages in avoiding ischemic–reperfusion injury, preventing immune rejection, and assisting the selection of suitable xenografts for liver transplantation. In the perfusion stage before transplantation, washing or cleaning the donor liver with human blood from recipients, could remove most of porcine leukocytes, porcine‐specific antibodies, and xenoantigens.^[^ ^21^, ^25^ ^]^ Additionally, blood used for perfusion contains many types of human lymphocytes, which may be used for testing cross‐species immunological incompatibilities and assessing or selecting suitable donor liver.^[^ ^21^, ^25^ ^]^ These advantages are crucial for avoiding xenogeneic immune rejection, but more importantly, NMP enables the possibility of ex vivo partial reprogramming. Lipid nanoparticles or adeno‐associated viruses encapsulating siRNA, CRISPR‐Cas, immunomodulators, and antibodies, introduced into the perfusate could circulate continuously allowing multiple passes through the liver cells and reprogramming the expression of xenoantigens and proinflammatory cytokines.^[^ ^21^, ^195^, ^196^ ^]^ In vitro partial reprogramming based on NMP is safer and easier for operation than in vivo. This method is effective to prevent the side effects of interaction with other organs.^[^ ^21^ ^]^ Moreover, it could accurately target the donor liver and avoid the off‐target effect of gene editing. These findings suggest that NMP would be an optimal platform for ex vivo partial reprogramming of a porcine xenograft before implantation.

### 6.2 Post‐Transplantation Techniques (Recipient Specific)

Immune rejection following xenotransplantation is fundamentally due to the differences of immune cells between porcine and human. The post‐transplantation techniques were aimed to induce immune tolerance in recipients. Advanced immunometabolism techniques could control the activation of immune response pathways and balance the ratio of cytotoxic lymphocytes to regulatory lymphocytes.

#### 6.2.1 Advanced Treg Induction Strategies Based on Metabolic and Epigenetic Reprogramming

The mTOR signaling pathway is a crucial regulatory pathway for Treg cell metabolism, and research on related inhibitors has long been a hot topic in inducing immune tolerance. As mentioned before, rapamycin, a classic inhibitor of mTOR protein, has been widely used in clinical practice for organ transplantation.^[^ ^197^ ^]^ However, long‐term use of such drugs increases the risk of potential complications such as opportunistic infections and tumors. To improve the safety and controllability of inducing immune tolerance, researchers have attempted to use novel low‐toxicity mTOR inhibitors, such as everolimus and RapaLink‐1, to induce immune tolerance during organ transplantation.^[^ ^150^, ^151^ ^]^ The third‐generation mTOR inhibitor RapaLink‐1 performs better than rapamycin in inhibiting the proliferation of effector T cells and could significantly prolong the survival time of transplants.^[^ ^150^ ^]^ Mechanistically, the improved rejection caused by RapaLink‐1 is related to the decrease in p4EBP1 in T cells, leading to an increase in Treg cells and a decrease in Th1 and Th17 cells.^[^ ^150^ ^]^ In addition to mTOR inhibitors, directly adding metabolic pathway intervention drugs or nutrients, such as 1,25‐dihydroxy vitamin D3, retinoic acid, and aminooxy acetic acid, could also induce T cell metabolic reprogramming during organ transplantation. Short‐term treatment with 1,25‐dihydroxy vitamin D3 and mycophenolic acid (MMF) increased the number of draining lymph nodes and spleen CD4+CD25+‐expressing CD152+ Tregs in tolerant mice.^[^ ^198^, ^199^ ^]^ Compared to untreated groups (<23 days), the treated group extended the survival time of heart and pancreatic transplants (>100 days).^[^ ^198^, ^199^ ^]^

Epigenetic modifications are crucial for Foxp3 gene expression and Treg cell metabolic reprogramming. Drugs targeting histone acetylation enzymes HDACs in Treg cells, such as SAHA, KA1010, TSA, and EX‐527, have significant potential in inducing immune tolerance in organ transplantation. In the transplant environment, HDAC inhibitors suppress transplant rejection and increase the number of CD4+ Tregs in transplants and secondary lymphoid tissues. SAHA targets HDAC6 or class I HDACs, increasing the ratio of Tregs to effector T cells by inducing apoptosis in effector T cells. KA1010 (a HDAC6 inhibitor, 160 mg for 28 days) increased the number of CD4+ Tregs but reduced the total number of CD4+ T cells, promoting the survival of mouse skin transplants (>60 days).^[^ ^200^ ^]^ Short‐term treatment with the HDAC9 inhibitor TSA and rapamycin promoted the conversion of CD4+CD25− to CD4+CD25+ Tregs in vivo. Interestingly, C57BL/6 recipients of BALB/C heart or pancreatic transplants who received combined treatment with TSA and rapamycin could tolerate secondary donor transplants (100 days).^[^ ^201^ ^]^ In addition to histone acetylation modification, the methylation modifications of histone and DNA are also related to the proliferation and differentiation speed of Treg cells. Supplementing specific metabolic intermediates, such as glutathione, all‐trans retinoic acid (atRA), alpha‐ketoglutarate, 2‐hydroxyglutarate (2‐HG), succinate, and fumarate, could regulate or change the activity of DNA methyltransferases, histone methyltransferases, DNA demethylases (Tets), and histone demethylases (HDMs), altering the epigenetic status of Treg cells. For example, adding glutathione could limit serine supply, inhibit the integration of one‐carbon units provided by serine metabolism into the methionine cycle for SAM generation, reduce histone and DNA methylation, and increase Foxp3 expression.^[^ ^202^ ^]^ Tets and HDMs are α‐KG‐dependent dioxygenases that use α‐KG and oxygen as substrates.^[^ ^203^, ^204^ ^]^ Succinate, fumarate, and 2‐HG are metabolic derivatives similar to α‐KG, acting as competitive inhibitors of these α‐KG‐dependent dioxygenases.^[^ ^203^, ^204^ ^]^ These metabolic products can be obtained from glucose or glutamine and participate in both synthetic and breakdown metabolism.^[^ ^204^ ^]^ Therefore, metabolic interventions involving changes in these metabolites regulate the activity of Tets and HDMs, thereby modulating the activation, differentiation, and function of Treg cells. Inhibiting glutamine conversion to α‐KG could prevent 2‐HG production, reduce methylation of Foxp3 gene loci, and increase Foxp3 expression.^[^ ^205^ ^]^ Consistent with this, Treg cells lacking isocitrate dehydrogenase (IDH) 1 and IDH2, enzymes that catalyze the reduction of α‐KG to 2‐HG, reducing the methylation levels of the Foxp3 promoter and CNS2 region.^[^ ^205^ ^]^

#### 6.2.2 CAR‐Treg Immunotherapy

**Figure 4:**
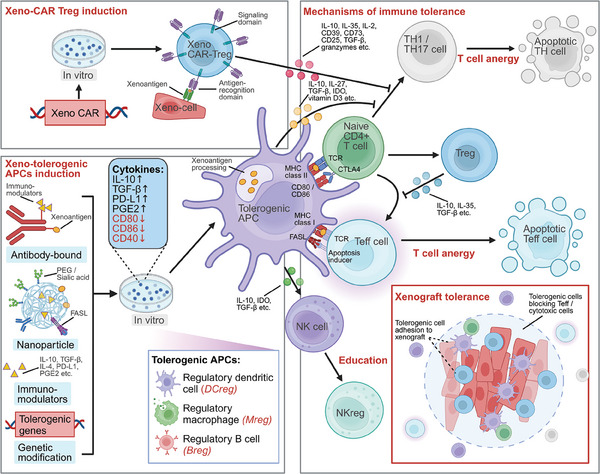
Advanced strategies for induction of xenoantigen‐specific tolerogenic cells. Tolerogenic cells consist of chimeric antigen receptor (CAR) regulatory T cells (Tregs) and tolerogenic antigen‐presenting cells (APCs), which contain xenoantigens originating from the donor porcine. These cells could be induced in vitro through gene editing, nanoparticles conjugated with xenoantigens and immunomodulators, and antibody‐based or cell‐based approaches. Tolerogenic cells could rapidly localize and aggregate around the xenograft, forming an immunotolerant “shield” that blocks effector T cells (Teffs) or proinflammatory cells from approaching the xenograft. After adhesion to the xenograft, tolerogenic cells reduce the expression of proinflammatory markers, including CD80, CD86, and CD40, and promote the production of tolerogenic molecules such as IL‐10, TGF‐β, inhibitory indoleamine 2,3‐dioxygenase (IDO), FAS ligand (FASL), programmed cell death ligand 1 (PD‐L1), and prostaglandin E2 (PGE2). These molecules assist tolerogenic cells in inducing naive CD4+ T cells to differentiate into Tregs, suppressing the generation of T helper cells (Th1 or Th17), inducing proinflammatory T cell anergy and ablation, and educating natural killer cells (NK cells) to apoptosis or kill cytotoxic cells. Abbreviations: PEG, polyethylene glycol; NKreg, regulatory NK cells. Adapted with permission from BioRender. Kelai, W. (2024).

CAR‐Tregs, derived from traditional CAR‐T technology, are mainly used to treat autoimmune diseases and resist immune rejection reactions (**Table** **4** and **Figure** **4**). Over the past decade, CAR‐Treg research has developed rapidly and has been applied in allogeneic transplantation.^[^ ^107^ ^]^ Traditional methods for generating antigen‐specific Tregs mainly rely on expansion with APCs and specific antigens or engineering Tregs with TCRs.^[^ ^206^ ^]^ However, the efficiency of expanding Tregs with APCs is low, and Tregs engineered with TCRs (TCR‐Tregs) are still restricted by MHC, limiting their modular application for different patients.^[^ ^206^ ^]^ Unlike traditional TCR‐Tregs, T cells expressing CARs bypass MHC restrictions when activated and have lower dependence on IL‐2.^[^ ^206^ ^]^ CARs activate Tregs leading to the upregulation of key regulatory Treg function proteins such as CTLA‐4, LAP, and GARP.^[^ ^207^ ^]^ Additionally, CAR‐Tregs could localize and aggregate around transplants more rapidly than TCR‐Tregs, which are crucial for protecting transplants, preventing transplant rejection, and inducing transplant tolerance. The main cause of allogeneic transplant rejection is attributed to incompatible donor and recipient HLA receptors, and about one‐fourth of transplant surgeries where the donor and recipient are HLA‐A2 positive and HLA‐A2 negative, respectively, could benefit from HLA‐A2 CAR Tregs.^[^ ^208^ ^]^ A plethora of research projects have outlined the spectrum of anti‐HLA‐A2 CARs (A2‐CARs) for Tregs and evaluated the in vitro and in vivo effects of human A2‐CAR Tregs.^[^ ^209^, ^210^, ^211^, ^212^ ^]^ A2‐CARs altered the specificity of nTregs but did not change their regulatory phenotype and epigenetic stability. Tregs activated by A2‐CARs showed stronger cell proliferation and upregulation of the CD39 effector molecule and inhibited the proliferation of allogeneic effector T (Teff) cells in vitro compared to unmodified nTregs or control CAR‐Tregs.^[^ ^210^ ^]^ Furthermore, in NOD.Rag1nullIL‐2gc (NRG) mice receiving A2‐CAR Tregs or control nTregs plus HLA‐A1+ PBMC (responders) and irradiated HLA‐A2+ PBMC (stimulants), A2‐CAR Tregs were more effective in inhibiting delayed hypersensitivity reactions compared to unmodified nTregs or control CAR‐Tregs, as measured by ear thickness.^[^ ^210^ ^]^

**Table 4:** Application of cell immunotherapy approaches on xenotransplantation.

Target tissue/condition	Type	Donor	Recipient	Antigen specificity	Induction agents	Outcomes	Refs.
Skin/islet cells	Xeno‐CAR Tregs	Porcine	Humanized mice	SLA‐specific scFvs	Absent	Xeno‐CAR Tregs completely prevented rejection of allogeneic target cells, porcine skin, and islets in reconstituted humanized mice.	[213]
Skin/islet cells	SLA‐CAR Tregs	Porcine	Humanized mice	SLA*0401 molecule	Absent	The modified CAR‐Tregs extended graft longevity.	[214]
Xenogeneic GVHD	HLA‐A2‐CAR Tregs	Human HLA‐A2‐positive PBMCs	Immunodeficient mice	HLA‐A2	Absent	Human anti‐HLA‐A2 CAR Tregs were better than polyclonal Tregs at inhibiting xenogeneic graft‐versus‐host disease in immunocompromised mice reconstituted with HLA‐A2‐positive human PBMCs.	[207]
Skin	HLA‐A2‐CAR Tregs	Human	Immunodeficient mice	HLA‐A2	Absent	HLA‐A2 CAR Tregs protected the HLA‐A2+ allografts more potently than polyclonal Tregs.	[209]
Xenogeneic GVHD	Induced DCregs	Porcine endothelial cells	Human CD8+ T cells	Various xenoantigens	Rapamycin and IL‐10	Both rapamycin‐ and IL‐10‐induced DCregs caused significantly less IFN‐γ production and human NK cell degranulation in response to porcine endothelial cells.	[258]
Islet cells	Induced DCregs	Rat	Diabetic mice	Various xenoantigens	IL‐10	Xenograft survival of rat islets in diabetic mice co‐transplanted with autologous murine IL‐10 DC was significantly prolonged from 12 to 21 days, without additional immunosuppressive treatment.	[259]
Islet cells	CD86‐silenced‐DCregs	Rat	Diabetic mice	Various xenoantigens	Absent	CD86‐silenced‐DCs induced immune tolerance of rat xenoislets in recipient diabetic mice with upregulated peripheral blood CD4+CD25+T cells.	[260]
Skin	IDO‐expressing fibroblasts	Human	Rat	Various xenoantigens	Absent	Indoleamine‐2,3‐dioxygenase (IDO) inhibits the xenoantigenic response and prolong survival time of skin xenografts through suppression of xenoantigen‐reactive proliferation of T cells and blocking of xenograft infiltration.	[261]
Xenogeneic GVHD	IDO‐expressing Mregs	Porcine PBMCs	Human PBMCs	Various xenoantigens	Macrophage colony‐stimulating factor (M‐CSF) and IFN‐γ	Induced Mreg with a phenotype of CD14lowCD16−/lowCD80lowCD83−/lowCD86+/hiHLA‐DR+/hi were capable of suppressing proliferating human PBMC, CD4+, and CD8+ T cells, even at a higher responder: Mreg ratio of 32:1 in a pig‐human xenogeneic MLR	[262]
Xenogeneic GVHD	CD200R+ Mregs	Porcine endothelial cells	Human CD200 cells	Various xenoantigens	Absent	The human CD200 cells inhibits the xenoantigenic rejection responses by CD200R+ Mregs, and hence, the creation of transgenic hCD200 pigs is suggested to attract for prevention of the macrophage cell‐induced rejection during xenografts transplantation	[263]

GVHD: graft‐versus‐host disease; SLA: swine leukocyte antigens; HLA: human leukocyte antigens; scFvs: single‐chain variable fragments.

In recent years, researchers have gradually explored the application of CAR‐Tregs in the field of xenogeneic transplantation tolerance (Table 4 and Figure 4).^[^ ^213^, ^214^ ^]^ Although the preparation system for CAR‐Tregs used in xenogeneic transplantation is not yet perfect, studies have shown that SLA CAR‐Tregs targeting SLA and xenogeneic‐specific CAR‐Tregs (Xeno CAR‐Tregs) targeting SLA‐specific single‐chain antibodies (scFvs) have potent immunomodulatory effects and could effectively prolong the survival time of transplants.^[^ ^213^, ^214^ ^]^ Compared to unmodified nTregs, SLA CAR‐Treg or Xeno CAR‐Treg significantly enhanced the ability to control strong immune responses in vitro and in vivo.^[^ ^213^, ^214^ ^]^ In the absence of any immunosuppressants, SLA CAR‐Tregs or Xeno CAR‐Tregs could perfectly prevent rejection of human transplants in a mouse model (NRG mice) and induce transplant‐specific tolerance.^[^ ^213^, ^214^ ^]^ If the issue of survival of CAR‐Treg cells after infusion is effectively addressed, these improved CAR‐Tregs will play a crucial role in human xenogeneic organ transplantation.^[^ ^213^, ^214^ ^]^

#### 6.2.3 Tolerogenic Antigen‐Presenting Cells Immunotherapy

In addition to CAR‐Treg therapy, cellular therapies for transplant tolerance, including tolerogenic or regulatory dendritic cell therapy (DCreg) and macrophage therapy (Mreg), are developing most rapidly (Table 4 and Figure 4).^[^ ^215^ ^]^ DCs are specialized APCs with specific functions in inducing immune tolerance mediated by T cells.^[^ ^215^ ^]^ Inhibitory molecules on tolerogenic DCs, such as programmed cell death ligand 1 (PD‐L1) and PD‐L2, bind to programmed cell death 1 (PD1) on T cells, promoting the differentiation of Foxp3+ Treg cells by downregulating phosphorylated AKT, mTOR, S6, and ERK2 while simultaneously upregulating phosphatase PTEN.^[^ ^216^ ^]^ Additionally, secreted factors that DCs release, such as TGF‐β, IL‐10, and IL‐27, promote Treg cell differentiation.^[^ ^216^ ^]^ There are fewer reported preparation schemes for human xenogeneic transplantation DCreg, but numerous schemes for generating DCreg have been described in animal models and human allogeneic transplantation (Table 4 and Figure 4).^[^ ^107^, ^215^ ^]^ Typically, peripheral blood mononuclear cells are separated from leukocyte products by washing and then cultured with granulocyte‐macrophage colony‐stimulating factor and IL‐4, as well as biological agents such as rapamycin, dexamethasone, vitamin D3, or anti‐inflammatory cytokines such as IL‐10.^[^ ^107^, ^215^ ^]^ The DCreg required for organ transplantation could be derived from the donor or the recipient. Donor‐derived DCreg cells could immediately present donor antigens to host T cells upon infusion, but these allogeneic cells are challenging to maintain long‐term survival after infusion.^[^ ^107^, ^215^ ^]^ In contrast, autologous DCreg induced by alloantigen are more operationally feasible. Injecting autologous DCreg before rat heart transplantation experiments could prolong the survival time of heart transplants and induce donor‐specific immune tolerance.^[^ ^107^, ^215^ ^]^ Trials of human living kidney transplantation have also shown that injecting autologous DCreg is feasible and safe, and injected DCreg could maintain their ability to induce immune tolerance for a long time.^[^ ^107^, ^215^ ^]^

#### 6.2.4 Targeting Age‐Specific Changes in T Cell Metabolism

If all T cells flowing into the transplanted liver were exhausted, the recipient would be at risk of viral infections, such as cytomegalovirus and hepatitis C virus. Instead of inducing T cell anergy, it is preferable to age T cells through metabolic reprogramming. Compared with young mice and humans, the activated CD4+ T cells from old ones failed to exhibit adequate metabolic reprogramming, leading to compromised metabolic pathways, particularly OXPHOS, glycolysis, and glutaminolysis.^[^ ^217^, ^218^, ^219^ ^]^ It makes aged mice or humans more prone to accept transplanted grafts.^[^ ^217^, ^220^ ^]^ Treating CD4+ T cells from young mice and humans with 6‐diazo‐5‐oxo‐l‐norleucine (DON), a glutaminolysis inhibitor, in combination with an inhibition of glycolysis (2‐deoxy‐d‐glu‐cose, 2‐DG) and OXPHOS (metformin), significantly reduced IFN‐γ production, dampened Th1‐ and Th17‐driven immune responses, prolonged the survival time of fully mismatched skin grafts.^[^ ^217^ ^]^ Remarkably, immunosuppressive capacities of the combination treatment (DON, 2‐DG, and metformin) were specific to CD4+ T cells rather than CD8+ T cells. Targeting the age‐specific metabolic reprogramming of CD8+ T cells did not alter transplant outcomes in either young or aged recipients.^[^ ^217^ ^]^

#### 6.2.5 Costimulation Blockade

The activation of T cells relies on co‐stimulation signaling pathways, which involve the interaction between costimulation molecules on the surface of APCs and their corresponding receptors on T cells. Blockading these costimulation signals could terminate T cell activation and prevent immune rejection in organ transplantation. The pathways of CD28/CTLA‐4/B7 (CD80/CD86) and CD40/CD40L (CD154) are the main components of co‐stimulation interactions. CD28 is homologous to CTLA‐4 and competitively binds to B7 or CD28L (CD80/CD86).^[^ ^221^ ^]^CD28 inhibitors, as the potential alternative immunosuppressive drugs, could directly bind to CD28 without interfering with the normal immunosuppressive signals mediated by CTLA‐4.^[^ ^222^ ^]^ Novel pegylated anti‐CD28 antibodies, such as FR104 and lulizumab, have been shown to be safe and well‐tolerated in healthy subjects and could effectively prevent rejection in NHP cardiac and kidney allotransplantation.^[^ ^223^, ^224^ ^]^ CTLA‐4 has a much higher binding affinity for B7 molecules compared to CD28. CTLA‐4‐Ig (a fusion protein of CTLA‐4 and IgG1), as a competitive inhibitor of the CD28/B7 interaction, has been widely used in the treatment of autoimmune diseases and in preventing immune rejection of allotransplantation.^[^ ^225^, ^226^ ^]^ However, the activity of CTLA‐4‐Ig to inhibit B7‐2 (CD86) is significantly less than that of endogenous CTLA‐4, which may lead to incomplete blockade of B7‐mediated T cell activation in vivo.^[^ ^227^ ^]^ Consequently, in NHP xenogeneic kidney transplantation experiments, recipients treated with CTLA‐4‐Ig still experienced antibody or cell‐mediated rejections during the early stage of post‐transplantation (2–3 weeks).^[^ ^227^ ^]^ Other than CD28/CTLA‐4/B7, the CD40/CD40L pathway plays a crucial role in T cell‐mediated activation of dendritic cells and macrophages, as well as T cell‐dependent humoral immune responses.^[^ ^228^ ^]^ Inhibiting the CD40/CD40L interaction will suppress the activation of B cells and APCs, and the following activation or differentiation of T cells.^[^ ^228^ ^]^ In preclinical models of pig‐to‐NHP transplantation, the use of anti‐CD40 or anti‐CD154 antibodies to block the CD40/CD40L pathway has effectively prolonged the rejection‐free survival of xenografts.^[^ ^227^, ^229^ ^]^ Since CD154 is expressed not only on T and B cells but also on activated platelets, traditional anti‐CD40L antibodies (such as HU5C8 or ABI793) could lead to thromboembolic events in recipients.^[^ ^230^, ^231^ ^]^ It is believed that thrombosis is related to the Fc region of the anti‐CD154 antibody.^[^ ^232^ ^]^ Through Fc region engineering, the novel anti‐CD154 antibody TNX‐1500 reduces binding to Fc‐γ receptor IIa, thereby effectively preventing adverse thromboembolic events.^[^ ^232^ ^]^ In xenotransplantation models, administration of TNX‐1500 could effectively block the CD40/CD154 pathway, significantly prolonging the survival of xenografts without causing thromboembolic events.^[^ ^233^ ^]^ These findings have proven the crucial role of costimulation blockade in xenotransplantation. Further improvement of medicines developed based on costimulation blockade may facilitate xenogeneic liver transplantation.

#### 6.2.6 Promote Immune Tolerance with Engineered Biomaterials

Engineered biomaterials potentially benefit to the xenograft by inducing immune tolerance after transplantation. Encapsulating immunosuppressants into biomaterial depots, such as synthetic biodegradable polymers, and micelles made with phospholipids or amphiphilic polymers, is a compelling strategy for precision pharmacokinetics to mitigate some of the known issues with systemic administration of these drugs.^[^ ^234^, ^235^, ^236^ ^]^ Conjugating immunosuppressive agents (mofetil, tacrolimus, etc.) to fatty acids to form lipid nanoparticles (LNPs) could prevent immune cell infiltration into transplanted liver graft and inhibit acute transplantation rejections.^[^ ^234^ ^]^ Rather than general release methods, biomaterial carriers could be engineered to respond target cells by designing their release profiles dictated by molecular dynamics involved in the pathology of disease or alloreactivity.^[^ ^234^ ^]^ To achieve immunoisolation, microencapsulation and ImmunoCloak techniques have been developed to encapsulate transplanted organs to concealing graft antigens. Hydrogels for wrapping transplanted organs have the advantages of strong histocompatibility and selective permeability against recipient IgG antibodies capable of complement activation and antibody‐mediated rejection, but not against smaller nutrients.^[^ ^234^, ^237^, ^238^ ^]^ Mice models for transplantation research have demonstrated the validity and feasibility of the encapsulation method with hydrogel.^[^ ^239^ ^]^ However, it is more effective for xenografts (pancreatic islets, ovaries, etc.) than allografts, since acute immune rejection of xenotransplantation is primarily mediated by host antibody.^[^ ^239^ ^]^

More importantly, biomaterials could deliver compounds that establish a self‐sustaining tolerogenic microenvironment for recipients through Treg induction or recruitment to achieve long‐lived operational tolerance. Polyethylene glycol (PEG) and poly(lactic‐*co*‐glycolic acid) (PLGA) are among the most commonly used synthetic polymers in immune tolerance induction. These materials are biocompatible, degradable, and could be chemically modified to facilitate various conjugation and network formation strategies.^[^ ^234^ ^]^ Biotinylated PEG microgels or biotin–PLGA scaffolds could be surface‐functionalized with bioactive signaling components, such as Fas ligand (FasL) and PD‐L1, which promote Treg conversion, effector T cell apoptosis, and extend the survival of transplanted grafts.^[^ ^240^ ^]^ Biomaterials could also boost Treg generation by using conjugating ligands of interest immunomodulators (IL‐10, TGF‐β, etc.) or mTOR inhibitors (sirolimus, everolimus, etc.) to carriers like glass beads using Staudinger ligation to azide groups.^[^ ^241^ ^]^ Another applicable strategy is to explore biomaterials to cotransplant tolerogenic APCs, which induce CD4+ T cells to anergy or an induced Treg state by presenting antigens with downregulated expression of critical CD80/CD86 costimulatory ligands.^[^ ^234^ ^]^ Compared with an unpredictable systemic infusion of tolerogenic APCs, biomaterials, such as PEG–methacrylate (PEG–MAA) hydrogel scaffolds, are better for localizing tolerogenic APCs into target organs.^[^ ^242^ ^]^ Previous studies have demonstrated that the intrinsic surface chemistry and charge profile of PEG–MAA could promote the adsorption of a specific milieu of complement proteins, leading to immune signaling pathway modulation for the recruitment of neutrophils and subsequent polarization of APCs.^[^ ^242^ ^]^ Further improvement of these biomaterials may have far‐reaching implications for xenotransplantation

## 7 Conclusions and Future Prospects

Xenotransplantation is one of the most important solutions for the shortage of liver organs. However, at present, the xenografts transplanted into human patients still experience rejections and complications, making long‐term survival unfeasible. The bottleneck in extending the survival time of xenografts lies in the lack of information on the metabolic process of xenoantigens production and the regulatory role of human metabolism on immune responses after transplantation. Metabolic differences between pigs and humans, such as the pathways for the generation of Gal, Neu5Gc, and Sda/Cad, are directly responsible for the appearance of heterologous antigens. Through the modification of immunometabolism pathways by gene editing techniques, we have been able to reduce or prevent the occurrence of acute immune rejections dramatically. While the mechanisms of production and regulation of many metabolites or antigens, such as GABARAPL1, COX2, and HMGB1, have still not been fully understood. To better investigate that, advanced multiomics techniques (spatiotemporal omics, metabolomics, epigenomics, single‐cell omics, etc.) should be employed in the comparative research between humans and pigs.

High‐throughput multiomics techniques are also important for detecting the changes in liver immunometabolism after transplantation. After xenotransplantation, pig livers will replace those of human patients to maintain the stability of metabolism and immune system. The incompatibility of immunometabolism between humans and pigs induces rejection, inflammation, coagulation dysfunction, and liver failure. We must avoid the severe “immune storm” while ensuring that human patients do not lose their normal immune function. Traditional immunosuppressants are hard to achieve due to their toxicity and side effects. Compared with them, techniques that target immunometabolism pathways to induce immune tolerance, such as CAR‐Treg and T‐cell aging, are more promising. Due to controllable characteristics, low toxicity, and minimal side effects, these techniques have been widely studied in allotransplantation and are gradually being applied in clinical practice. However, in xenotransplantation, their application is limited due to the technical barriers in the preparation of tolerogenic cells by induction of “xenogenes.” Further improvement of current immunometabolism therapies, including the modification of biomaterial scaffolds to enhance the localization and enrichment of tolerogenic immune cells, is required. In addition, we should emphasize the integration of research on pre‐transplantation and post‐transplantation. Separated research makes it difficult to assess the safety and reliability of gene‐edited livers accurately. The profile of immunometabolism after liver xenotransplantation may be helpful to find the potential risks behind knock‐out porcine genes or knock‐in human genes.

## Conflict of Interest

The authors declare no conflict of interest.

## Author Contributions

S.D. and Y.Z. contributed equally to this work. S.D., Y.Z., S.S., and C.L. wrote the original draft preparation. C.Q. contributed to supervision. All authors have read and agreed to the published version of the manuscript.
